# Resistance in the Genus *Spodoptera*: Key Insect Detoxification Genes

**DOI:** 10.3390/insects12060544

**Published:** 2021-06-11

**Authors:** Frédérique Hilliou, Thomas Chertemps, Martine Maïbèche, Gaëlle Le Goff

**Affiliations:** 1 Université Côte D’Azur, INRAE, CNRS, ISA, F-06903 Sophia Antipolis, France; frederique.hilliou@inrae.fr; 2 Institut D’Ecologie et des Sciences de L’Environnement de Paris, Sorbonne Université, CNRS, INRAE, IRD, iEES-Paris, F-75005 Paris, France; thomas.chertemps@sorbonne-universite.fr (T.C.); martine.maibeche@sorbonne-universite.fr (M.M.)

**Keywords:** resistance, *Spodoptera*, cytochromes P450, carboxyl/cholinesterases, glutathione S-transferases, ATP-binding cassette transporters

## Abstract

**Simple Summary:**

The moth larvae are among the most damaging pest species on crops worldwide. In this review, we focus on the genus *Spodoptera*, which can feed on many crops such as rice, cotton or corn. The massive use of insecticides to control these insects has led to the development of resistance. Here, we aim to compare the resistance mechanisms of four species (*Spodoptera exigua*, *Spodoptera frugiperda*, *Spodoptera littoralis* and *Spodoptera litura*) and highlight the role of enzymes and transporters in resistance to help us understand the molecular basis of their origin.

**Abstract:**

The genus *Spodoptera* (Lepidoptera: Noctuidae) includes species that are among the most important crop pests in the world. These polyphagous species are able to feed on many plants, including corn, rice and cotton. In addition to their ability to adapt to toxic compounds produced by plants, they have developed resistance to the chemical insecticides used for their control. One of the main mechanisms developed by insects to become resistant involves detoxification enzymes. In this review, we illustrate some examples of the role of major families of detoxification enzymes such as cytochromes P450, carboxyl/cholinesterases, glutathione S-transferases (GST) and transporters such as ATP-binding cassette (ABC) transporters in insecticide resistance. We compare available data for four species, *Spodoptera exigua*, *S. frugiperda*, *S. littoralis* and *S. litura*. Molecular mechanisms underlying the involvement of these genes in resistance will be described, including the duplication of the CYP9A cluster, over-expression of GST epsilon or point mutations in acetylcholinesterase and ABCC2. This review is not intended to be exhaustive but to highlight the key roles of certain genes.

## 1. Introduction

The genus *Spodoptera* (Lepidotera: Noctuidae) contains some of the most important insect crop pests, many of which are highly polyphagous species, able to feed on more than 100 host plants including maize, rice, cotton (e.g., *Spodoptera litura* (Fabricius) [1]). They are present on all continents and their potential invasiveness has been highlighted in recent years, notably with the species *S. frugiperda* (J.E. Smith). Native to the American continent, it was detected in Africa in 2016 [2] and has since invaded Asia and Australia (CABI, Wallingford, UK, 2021). With its flight capabilities [3,4] and under favorable climatic conditions, its invasion of Europe in the near future seems inevitable.

The control of these pests requires the massive use of insecticides. They have developed resistance to all chemical families and three of the four *Spodoptera* species present in the Arthropod Pesticide Resistance Database are in the top 15 most resistant arthropods on the planet: *S. litura*, *S. frugiperda* and *S. exigua* (Hübner) [5].

Table 1 shows the available data on the molecules for which these three species, as well as *S. littoralis* (Boisduval) have developed resistance. These insects have developed resistance to all the chemical families: organophosphates, carbamates, pyrethroids but also for a more recent family such as diamides. There are usually two main mechanisms in insecticide resistance, either target modification or mechanisms that reduce the amount of insecticide reaching the target (reduced penetration, sequestration or intervention of detoxification enzymes). Detoxification involves enzymes that catalyze successive reactions to make the insecticidal molecule less toxic and more easily excreted from the body. Cytochromes P450 (P450) and carboxyl/cholinesterases (CCE) are phase I enzymes that catalyze oxidation, hydrolysis and reduction. Glutathione S-transferases (GST) are phase II enzymes and catalyze the addition of a group such as glutathione. This step is called conjugation and is followed by phase III excretion involving ATP-binding cassette (ABC) transporters. These proteins use the energy of ATP hydrolysis to transport substrates across lipid membranes. The resistance caused by the enzymes involved in these three phases implies either a modification of their level of expression or their catalytic activity. The molecular process behind this can be of several kinds. A point mutation in the sequence of a gene can modify the catalytic activity of the enzyme. For example, in the mosquito *Anopheles funestus*, the increased activity of GSTe2 is due to a point mutation (L119F) that leads to an increase in the accessibility of the active site, allowing high resistance to DDT [6]. Overexpression is one of the other commonly demonstrated mechanisms, involving duplications, amplifications or cis-or trans-regulations [7]. A well-known example of gene duplication is the case of *CYP6G1* in *Drosophila melanogaster*. The overexpression of this gene in multi-insecticide resistant populations is due to duplication as well as insertion of transposable elements in its promoter [8]. In the aphid *Myzus persicae*, it is a gene amplification of one or two F4 and FE4 esterases that is at the origin of the resistance to organosphosphates, carbamates and pyrethroids [9]. Up to 80 copies of the same gene are found in some resistant aphids. The esterase in this case represented up to 3% of the total proteins [10]. A recent example illustrates cis and trans regulation in a chlorpyrifos-resistant strain of *S. exigua* [11]. The authors showed that the resistance was mainly due to the overexpression of a P450, CYP321A8, a P450 capable of metabolizing chlorpyrifos, cypermethrin and deltamethrin. This overexpression is due to both constitutive overexpression of the transcription factors Cap’n’collar isoform C (CncC) and Maf (Muscle aponeurosis fibromatosis, trans-regulation), major regulators of detoxification in insects [12], and to a mutation in the promoter of this P450. The mutation creates a cis-regulatory element that promotes binding of a Knirps nuclear receptor.

This review highlights some examples that demonstrate the involvement of each of the four families of detoxification genes (P450, CCE, GST and ABC) in insecticide resistance and the molecular mechanisms identified for pests of the genus *Spodoptera*. We have chosen to focus on specific genes that we consider emblematic rather than an exhaustive review of their roles.

## 2. Phase I: Functionalization

Once a xenobiotic enters the cell, it is processed by enzymes that make it functional. The phase I enzymes carry out oxidation reactions, hydrolysis, etc., making the metabolite more polar and more easily excreted.

### 2.1. Cytochrome P450s

Cytochrome P450 genes (CYP) constitute one of the largest gene families with representatives in nearly all living organisms [13]. CYPs catalyze a large number of reactions including hydroxylation, epoxidation, oxidation [14]. In insects, CYPs are involved in endogenous metabolism such as the biosynthesis of ecdysteroid [15] or the production of hydrocarbon [16], as well as in detoxification mechanisms with the metabolization of xenobiotics (e.g., plant secondary metabolites and insecticides). Their number varies from 36 in the louse *Pediculus humanus* [17] to 196 in the mosquito, *Culex quinquefasciatus* [18]. Nomenclature has been proposed based on sequence homology [19,20]. P450s belong to the same family designated by an Arabic numeral when they share 40% identity and to the same subfamily designated by capital letters when this percentage is higher than 55%. The nomenclature was revised and the notion of clan was proposed as a higher level of classification to take into account the increasing number of available sequences [21]. Initially, the arthropod P450s were divided into four clans, but a recent study brings this number to 6 with the historical clans, clan 2, clan 3, clan 4, mitochondrial clan to which clans 16 and 20 were added [22]. Clans 3 and 4 are the two main clans known to have P450s involved in resistance mechanisms, but not exclusively, for example, *CYP12D1* from *D. melanogaster* which belongs to the mitochondrial clan is over-expressed in resistant strains [23] and has been shown to confer resistance to DDT and dicyclanil [24]. We will highlight the role of some of P450s in insecticide resistance and evolution of the *CYP9A* subfamily within the four *Spodoptera* species.

#### 2.1.1. Phylogeny of *CYP9A*

**Figure 1:**
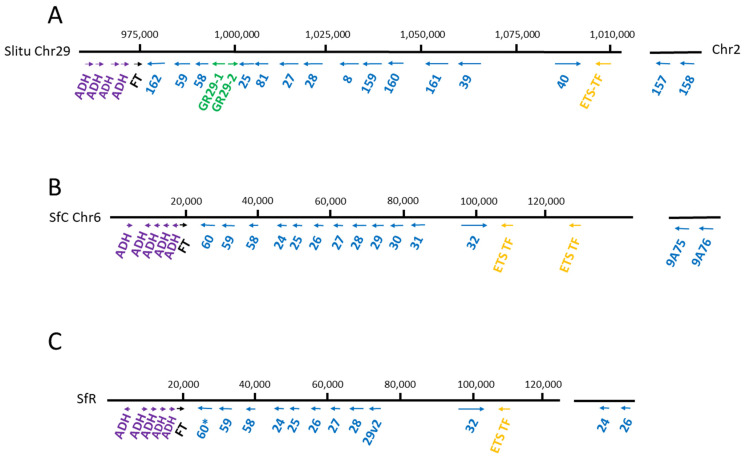
CYP9A cluster in the genome of *S. litura* (**A**), *S. frugiperda* corn strain (**B**) and *S. frugiperda* rice strain (**C**). Numbers in blue correspond to *CYP9A* genes. Arrows correspond to gene orientation, ADH: alcohol dehydrogenase, FT: fucosyl transferase, EST TF: E26 transformation specific transcription factor, * pseudogene.

*CYP9A* belongs to clan 3. In Lepidoptera, *CYP9As* were identified as a cluster in a comparative study of BAC sequences from three species *Bombyx mori*, *Helicoverpa armigera* and *S. frugiperda* [25] with the presence of four, five and nine genes respectively, showing the rapid evolution of the cluster. In the two more closely related species (*H. armigera* and *S. frugiperda* which diverged about ±20 MYA), only three orthologous pairs were identified [26]. The availability of the genome of the most closely related species in the genus *Spodoptera* enabled the evolution of this group to be assessed. In *S. frugiperda*, obtaining the chromosomally resolved genome of the maize strain helped identify 14 *CYP9A* genes (genome Version 6 [27]), 12 of which were in a cluster (Figure 1), i.e., three more than the nine genes previously identified in the BAC study, whereas 15 genes were identified in the rice strain (genome version 3 [28]). These numbers are very similar to those found in other species, 15 genes in *S. litura* (Figure 1) [1], 12 genes in *S. exigua* and 11 genes in *S. littoralis* (genome in progress, transcriptome available). In the latter, the number is potentially underestimated and improved genome resolution could lead to increases.

**Figure 2:**
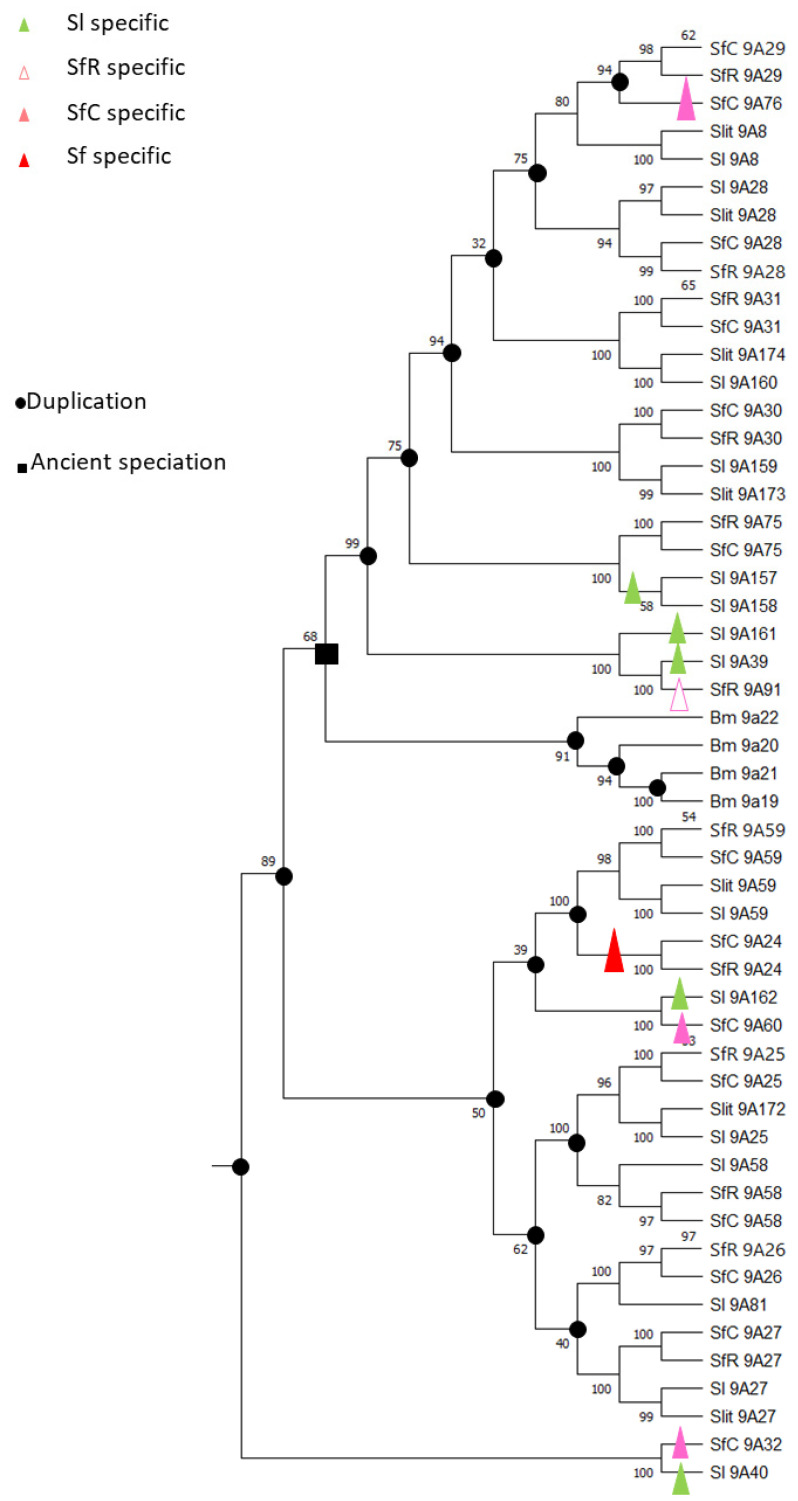
*CYP9A* subtree: ML phylogeny with bootstrap value. SfC: *S. frugiperda* corn strain, SfR: *S. frugiperda* rice strain, Sl: *S. litura*, Slit: *S. littoralis*, Bm: *B. mori*. Slit9A58, Slit9A81, and Slit9A40-like are missing from the tree. Black square: ancient speciation representing *B. mori* split from *Spodoptera* species. Black dot: duplication events.

Among these genes, eight were true orthologs, *CYP9A25*, *CYP9A26*, *CYP9A27*, *CYP9A28*, *CYP9A30*, *CYP9A31*, *CYP9A58* and *CYP9A59* in *S. frugiperda*, *S. litura* and *S. littoralis*, probably already clustered in their common ancestor (Figure 2). *CYP9A24* is only found in *S. frugiperda*. Some genes of the cluster are *S. frugiperda* corn strain specific (*CYP9A32*, *CYP9A60*, and *CYP9A76*), *S. frugiperda* rice strain specific (*CYP9A91*) or *S. litura* specific (*CYP9A39-157-158-161-162*). Orthologs of *CYP9A26*, *CYP9A59* and *CYP9A27* were also found in the genome of *S. exigua.*

The synteny of the CYP9 cluster is conserved between *S. frugiperda* (corn and rice variants) and *S. litura* (Figure 1) where the same genes, alcohol dehydrogenase (*ADH*), fucosyl transferase (*FT*) and E26 transformation specific (*ETS*, a transcription factor) were found on each side of the cluster. In *S. litura*, two gustatory receptors from the GR29 family are part of the *CYP9* cluster. They form a head to tail tandem between *CYP9A58* and *CYP9A25* and they replace *CYP9A24*. The GR29 family is specific to *Spodoptera* and based on the evolutionary history of *S. litura* and *S. littoralis* [29], we expect GR genes to be present in the *CYP9* cluster of *S. littoralis*. The mechanisms resulting in blooming in the *CYP9A* family in the *Spodoptera* complex still remain to be deciphered. Several origins are possible, one of which corresponds to transposable elements as they have been shown to be prevalent in the surrounding of CYP genes in Lepidoptera [25,30,31], *Drosophila* [32] or mosquito [33]. Other mechanisms include duplications (tandem, chromosome or genome duplications) and retropositions [26]. This *CYP9A* bloom likely provides *Spodoptera* species with a selective advantage and potentially diverse catalytic capabilities for each of these enzymes. Indeed, induction experiments show that within the *CYP9A* cluster, genes have their own induction profile depending on the xenobiotics used. For example, *CYP9A31* is the most induced gene in response to xanthotoxin treatment in both *S. frugiperda* and *S. litura* whereas *CYP9A28* is not induced by this molecule but by indole [34,35]. This suggests that within the cluster, each gene has its own catalytic competence unrelated to its position in the cluster or phylogeny as it has been suggested for the *CYP6AE* cluster in *H. armigera* [36]. Dermauw et al., (2020) suggested that there would be a selective advantage to keeping the cluster as a heritable unit, which would enable adaptation to new environments [22].

#### 2.1.2. Resistance through Over-Expression of *CYP9A*

We next examine CYP9As in the light of insecticide resistance: the first *CYP9A* was found in *Heliothis virescens* and named *CYP9A1* [37]. It was in a thiodicarb-resistant population of *H. virescens*. An elevated level of *CYP9A1* mRNA was detected in the resistant strain compared to the susceptible strain. Several subsequent studies have associated over-expression of certain *CYP9As* with insecticide resistance, but very few studies have gone so far as to demonstrate their involvement in insecticide metabolism. An example came from *H. armigera*, where pyrethroid resistance was associated with constitutive over-expression of P450s in the laboratory-selected YGF strain [38]. In this strain, *CYP9A12* and *CYP9A14* were over-expressed 433-and 59-fold, respectively, in the fat body. The functional expression of these two P450s in *Saccharomyces cerevisiae* demonstrated their capacity to metabolize a pyrethroid insecticide, esfenvalerate [39]. Elevated levels of *CYP9As* associated with resistance have also been shown in *Spodoptera* spp. In a recent study, a Brazilian population of *S. frugiperda* resistant to *Bacillus thuringiensis* (Bt) Cry1 toxins was tested against 15 insecticide molecules with different modes of action. In addition to resistance to Bt toxins, the Sf-Des strain had showed 14- and eight-fold resistance to deltamethrin and chlorpyrifos, respectively [40]. P450 activity was increased in the Sf-Des strain compared to the susceptible Bt strain. RNAseq experiments confirmed the over-expression of several P450s, including *CYP9As* and RT-qPCR analysis validated that a *CYP9A-like* was expressed more than 200-fold in the resistant strain. In another study on a laboratory-selected population of *S. frugiperda* with lufenuron, a *CYP9A-like* gene was over-expressed 45-fold [41]. In both of these studies, the cause of the over-expression of the *CYP9As* was not identified, whereas Gimenez et al., (2020) reported the existence of two copies of the full CYP9A cluster of in a Puerto Rican resistant strain (PR) of *S. frugiperda*. PR was resistant to deltamethrin and the use of a P450 synergist, piperonil butoxide (PBO) abolished the resistance, confirming the primary role of P450. The *CYP9A* cluster locus is under positive selection in this strain [27]. Several field-collected strains of *S. exigua* resistant to organophosphates, pyrethroids and diamides also exhibited an over-expression of *CYP9A*. For a chlorpyrifos-resistant strain, the resistance was mainly associated with *CYP321A8*, which is over-expressed and capable of metabolising insecticides, however monitoring the expression of 68 P450s by RT-qPCR showed significant over-expression of several *CYP9As* including *CYP9A11*, *CYP9A27*, *CYP9A97* and *CYP9A98* [11]. Are these genes also involved in resistance? This remains to be demonstrated. However, there is some evidence of their involvement in resistance, such as the RNAi knockout (KO) of *CYP9A98*, which makes *S. exigua* larvae more sensitive to deltamethrin [42], the knockout of *CYP9A10*, which makes them more sensitive to alpha-cypermethrin [43], and the knockout of *CYP9A105*, which increases mortality to deltamethrin, alpha-cypermethrin, and fenvalerate [44]. Similar results were also obtained in the species *S. litura*. Indeed, strains resistant to fenvalerate, beta-cypermethrin and cyhalothrin had their resistance reduced by the use of PBO [45]. RNAseq experiments for these field-collected Chinese strains (LF and NJ) showed over-expression of several P450s including *CYP9As*. *CYP9A40* was over-expressed 663.4- and 76.13-fold in LF and NJ, respectively, compared with the susceptible strain. Previous experiments showed that dsRNA silencing of *CYP9A40* increased the sensitivity of *S. litura* to deltamethrin [46]. To our knowledge, no data suggesting a link between insecticide resistance and *CYP9A* of *S. littoralis* are available at this time. There is sufficient evidence across closely related species to suggest a major role for *CYP9As* at least in pyrethroid resistance; however, the ability of these enzymes to metabolise insecticides remains to be demonstrated. The cause of the over-expression of these genes also remains to be identified. In one case, it is due to the duplication of the *CYP9A* cluster [27] while in the study of Hu et al., (2021) it can be assumed that the over-expression of the transcription factor CncC, already shown in several insects to be the major regulator of detoxification genes, leads to the over-expression of *CYP9A*.

### 2.2. Carboxyl/Cholinesterases (CCEs)

Insect carboxylesterases or carboxyl/cholinesterases (CCEs) have a wide range of physiological functions across living organisms, from metabolism of endogenous compounds (hormones, pheromones, neurotransmitters) to detoxification of various xenobiotics. They play a critical role in defense against various allelochemicals associated with plants and insecticides [47]. In particular, CCEs have been implicated in resistance to pyrethroids (PYRs), organophosphates and carbamates (CBs) in numerous pest species [48,49]. The mechanisms of esterase-mediated resistance involve either metabolic resistance or target site mutation [49]. Metabolic resistance can be based either on insecticide sequestration or insecticide hydrolysis: overproduction of CCEs through gene amplification or transcriptional up-regulation can lead to insecticide molecule sequestration without any (or very slow) hydrolysis of the insecticide, whereas point mutations can alter the catalytic properties of some CCEs, leading to increased hydrolysis towards insecticides. Target site resistance is due to mutation of acetylcholinesterases (AChEs) that renders them less sensitive to inhibition by insecticides.

Insect CCEs fall into three main functional groups, representing dietary/detoxification, hormone/semiochemical processing, and neuro/developmental functions [50]. A comprehensive phylogenetic analysis of CCE sequences isolated from *H. armigera*, *H. zea*, *B. mori* and *Manduca sexta* genomes has recently refined the CCE nomenclature in Lepidoptera [51]. The neuro/developmental class comprises seven clades (027 to 033) with generally catalytically incompetent proteins, excepted for AChEs (clade 027). The hormone/semiochemical processing class (clades 020 to 026) includes among others, juvenile hormone esterases, several pheromone-processing esterases, but also some CCEs (clade 026) previously associated with organophosphate (OP) resistance in Hemiptera (namely E4, FE4, [10]). Finally, the dietary/detoxification group (clades 001 to 019 and 034) is the most diversified: some enzymes have been implicated in semiochemical processing but most of them have been associated with dietary and detoxification functions, including insecticide resistance for some dipteran and hymenopteran CCEs (the α-esterases, reviewed in [47]). CCEs from this third group are the most abundant in Lepidoptera and in particular the three most numerous clades (001, 006 and 016) with species-specific radiations. Many clades in this third group are also Lepidoptera specific [51]. The total number of CCE genes identified so far in *S. litura* and *S. frugiperda* genomes were 110 and 96, respectively [1,28]. For *S. exigua*, we were able to retrieve 73 sequences from public database (GenBank:GCA 011316535.1). For *S. littoralis*, we have previously identified 30 CCE transcripts expressed in antennae [52,53,54]. This repertoire was supplemented with 26 new sequences by Walker et al., (2019) [55]. Searching in our RNAseq databases allowed us to identify an additional 19 CCE transcripts (Table S1), leading to a total of 65 SlitCCE sequences, which is likely underestimated compared to its sister species *S. litura*.

Although comprehensive complete repertoires of CCEs are now available in many lepidopteran species, including spodopterans, still very few CCE genes have been directly linked with insecticide resistance. Here, we will focus on some spodopteran CCEs for which a direct role in resistance is supported either by in vitro and/or in vivo approaches, i.e., enzymes both identified at the molecular level and directly involved in insecticide hydrolysis or insecticide susceptibility.

#### 2.2.1. Resistance through Over-Expression of CCE

As reviewed previously in Farnsworth et al., (2010), there is a strong correlation between insecticide resistance and higher esterase activities in the four *Spodoptera* species studied here [56]. Esterase activities toward artificial substrates in vitro (generally α-or β-naphthyl acetate) were compared between homogenates of susceptible and resistant strains, which allowed for the detection of both higher overall CCE activities or higher staining intensities of certain isozymes after analysis by native PAGE electrophoresis. For example, higher staining intensities of several esterases isozymes have been associated with OP and PYR resistance in *S. littoralis* [57], *S. litura* [58], *S. exigua* [59] and *S. frugiperda* [60]. The underlying biochemical mechanisms are not yet known, but it is hypothesized that increased sequestration of insecticides by CCEs and catabolism apply [56].

In lepidopterans, over-expression of CCE associated with insecticide resistance has been intensively studied in *H. armigera* [61,62,63], especially through proteomic and real-time polymerase chain reaction (RT-PCR) approaches. Most of the over-expressed CCEs identified at the molecular level belonged to the 001 clade, which is very diverse in this species, with 21 CCE001 genes. This clade also shows a large expansion in spodopterans, with 23 and 19 CCE001 genes annotated in *S. litura* [1] and *S. frugiperda* [28], respectively. We counted 14 CCE001 sequences in *S. littoralis* ([52,53,54,55] Table S1), a number which is probably again underestimated in the absence of genome annotation. In *S. litura*, some of these CCE001 genes were inducible by imidacloprid [1]. Most interestingly, knock-down of two SlituCEE001s (SlituCOE57 and SlituCOE58) by siRNA injection increased the sensitivity of *S. litura* larvae to imidacloprid when fed with an insecticide-containing diet [1]. This is the first demonstration of a direct link between CCE induction and insecticide sensitivity at the molecular level in a lepidopteran species. After BLAST (Basic Local Alignment Search Tool) searches, we found orthologous sequences of SlituCOE57 and SlituCOE58 in *S. littoralis* (SlitCXE50 and SlitCXE48, respectively, Table S1) and *S. frugiperda* (SfruCCE001n and SfruCCE001f, respectively, [55]), suggesting that they might play a similar role in insecticide resistance. In *S. litura*, all of these CCE001s were grouped into a large cluster on chromosome 2 [1], as previously observed for the CCE001 of *H. armigera* [51].

#### 2.2.2. Metabolic Resistance through Point Mutations of CCEs

Structural mutations in the active site of a carboxylesterase could result in a reduction in the ability of this enzyme to hydrolyze common carboxylesterase substrates (such as naphthyl acetate esters) but convert it to an OP hydrolase. This corresponds to the mutant ali-esterase mechanism [64]. Metabolic resistance by point mutation in CCE sequences has been observed for quite some time in several non-lepidopteran species, mostly in Diptera [49,65,66]. Two point mutations, G137D and W251L (or G151D and W271L as reported in Cui et al., (2011) following the sequence of *D. melanogaster* AChE [67]), were indeed found in field-resistant populations of several species, including *Lucilia cuprina* and *Musca domestica*, and involved in altering the substrate specificity of CCE, leading to increased activity towards OPs [65,66,67]. In addition to dipterans, another amino acid change at position 251 (W251G) was also found in an OP-resistant strain of the parasitoid wasp *Anisopteromalus calandrae* [68].

**Figure 3:**
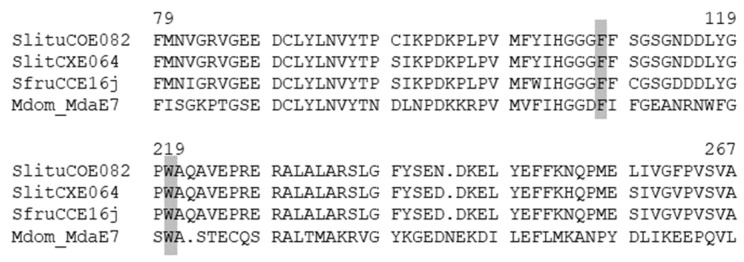
Alignment of two 50 amino acid sequence portions of SlituCOE082, SlitCXE64, SfruCCE16j and MdaE7 from *Musca domestica* (Rutgers diazinon-R resistant strain). The putative mutation positions G151D and W271L are shaded in grey. According to [67], the amino acid corresponding to G137D and W251L were G107 and W219 in *S. litura* sequence; for *S. litura* carboxyl/cholinesterase (CCE), the mutation tested in vitro was G151D. Genbank accession number, *S. frugiperda* (XP_035450257.1), *S. litura* (XP_022828113.1), *M. domestica* (AAD29685.1).

To the best of our knowledge, no point mutation-based resistance in CCE has been resolved at the molecular level in resistant Lepidopteran strains. However, an in vitro study conducted on CCEs from four insect orders including Lepidoptera, tested whether the change of substrate specificity associated with these two mutations could be a more general feature in OP-resistant insects [67]. The catalytic properties of seven CCEs mostly belonging to the dietary/detoxification group were analyzed, including one CCE from *S. litura* (GenBankEU783914). Recombinant mutant proteins were tested in vitro towards two OPs (paraoxon and chlorfenvinphos) and β-naphthyl acetate. For the seven enzymes tested, the G151D and W271L mutations conferred OP activity in 62.5% and 87.5% cases, respectively [67]. The same in vitro approach was developed more recently on eight *H. armigera* CCEs but with more contrasting results [62], with increased insecticide hydrolysis being observed only for some enzymes. However, for one esterase (HarmCCE001c), PYRs (fenvalerate and cypermethrine), hydrolysis was enhanced 14-fold after the leucine mutation. The *S. litura* CCE tested by [67] matched the SlituCOE082 genomic sequence identified later [1]. We found sequences orthologous to SlituCOE082 in *S. frugiperda* and *S. littoralis*, but not in *S. exigua*. SlituCOE82 presented 95.7% of amino acid identity with SlitCXE64 (Table S1) and 91.4% with SfruCCE16j [55], with residues conserved at the putative mutation positions (Figure 3).

**Figure 4:**
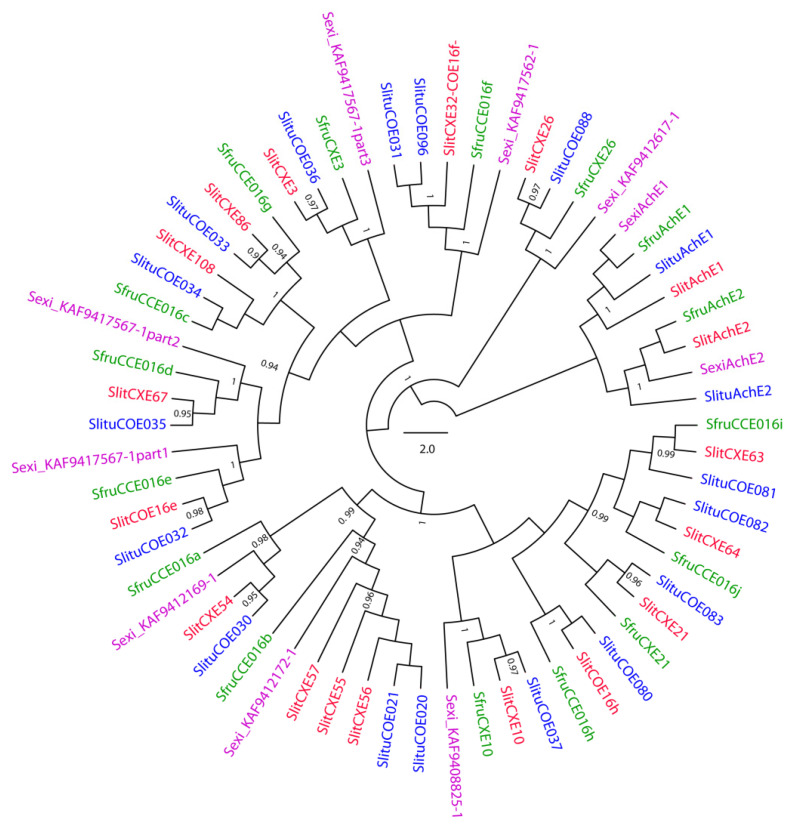
Maximum-likelihood phylogeny of spodopteran CCEs from Clade (016) and acetylcholinesterases (AChEs). The tree was built from amino-acid sequences of CCE repertoires of *S. littoralis* (branches colored in red), *S. frugiperda* Corn strains (green), *S. litura* (blue), *S. exigua* (purple). Sequences from [1,28] for *S. litura* and *S. frugiperda*, respectively. For *S. littoralis*, from [52,55] and new sequence from our RNAseq database (Table S1). For *S. exigua*, GenBank numbers were inserted the sequence name, SexiAChE1 (AZB49078.1) and SexiAChE2 (AZB49079.1). Likelihood-ratio test values are only indicated in the internal nodes defining orthogroups, when aLRT > 0.9. The scale bar represents 0.2 expected amino-acid substitutions per site.

SlituCOE082 belongs to the clade 016 which is also expanded in Lepidoptera [51]. It includes 16 genes in *S. litura*, 14 in *S. frugiperda*, eight in *S. exigua* and 16 in *S. littoralis* (including nine additional sequences from RNAseq data). Phylogenetic analysis of these 54 sequences (Figure 4) illustrates their diversification within the four Spodoptera species, and in particular the 1:1 orthologous relationship is clear for seven sequences from each species. In *S. litura*, the CCEs from in clade 016 showed a major expansion on chromosome 25, with a cluster of 12 adjacent sequences [1]. Six genes in this cluster showed induction by neonicotinoid insecticide, i.e., imidacloprid in *S. litura* larvae. In particular, SlituCOE82 is moderately induced by imidacloprid in Malpighian tubules [1].

To date, the presence of the G151D and/or W271L mutations in field-resistant spodopteran strains has not been found. However, although metabolic resistance by overproduction of CCE could induce broader resistance than qualitative mutation, the two studies discussed below suggest that point mutations could be a more common mechanism for insecticide resistance than expected. A systematic comparison of the corresponding CCE gene sequences in susceptible and resistant lepidopteran pest strains would be necessary to assess their precise role in resistance.

#### 2.2.3. Target Site Resistance through Point Mutations; the Case of Acetylcholinesterases

Acetylcholinesterase (AChE or ace) terminates nerve impulses by catalyzing the hydrolysis of acetylcholine in cholinergic synapses. Irreversible inhibition of AChE by CBs and OPs thus causes acetylcholine to accumulate in synapses and acetylcholine receptors to open permanently, resulting in insect death. Most insects have two *AChE* genes but only the gene expressed in the central nervous system (namely ace-1 or *AChE1*) is essential for synapse functioning [69]. All four *Spodoptera* species possess two *AChE* genes as expected (Figure 4) with a clear orthologous relationship and highly conserved sequences (more than 98% of amino acid identity). Target site resistance mediated by *AChE1* insensitivity to insecticides has been identified by biochemical approaches in several insect species, and subsequently elucidated at the molecular level for some species [49,69]. Sequencing of *AChE* genes from field-resistant strains and comparison with susceptible strains described several point mutations conserved across species, most of which modify the active site of the enzyme. Three substitutions (A201S, G227A and F290V, numbering corresponding to the mature enzyme of *Torpedo californica*) were first reported in CB- and/or OP-resistant strains of aphids (*A. gossypii* [70]), dipterans (*Bactrocera dorsalis* [71]) and lepidopterans (*Chilo suppressalis* [72] and in *P. xylostella* [73]).

**Figure 5:**
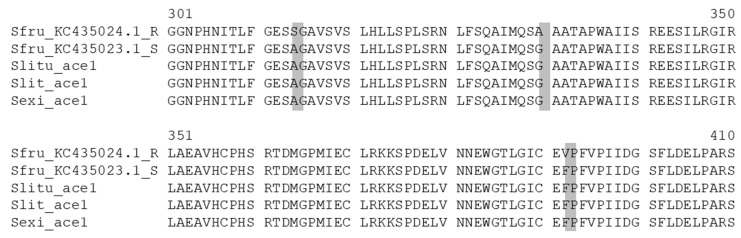
Alignment of a 120 amino acid sequence portion of AChE1 *S. frugiperda* susceptible (Genbank accession number KC435023.1) and resistant strain (KC435024.1) with orthologous sequences from *S. litura* (XP_022819835.1 = SlituCOE002), *S. littoralis* (Table S1) and *S. exigua* (AZB49078.1). The mutation positions are shaded in grey.

The involvement of acetylcholinesterases in OP/CB resistance has been demonstrated in biochemical approaches on *S. litura* resistant strains from Korea [74] or India [75,76]. Similarly, in *S. exigua*, the AChE enzyme of a carbamate resistant strain from California was found to be approximately 30-fold more insensitive to methomyl compared to the enzyme from the susceptible laboratory strain [77]. However, within Spodoptera species, molecular data on the AChE point mutation are mostly available for *S. frugiperda*. In this species, Yu et al., (2003) showed that acetylcholinesterase of a field strain collected from corn fields in Florida was clearly far less sensitive (up to 85-fold) than that of a susceptible strain to inhibition by CBs and OPs [60]. Kinetic data also showed that the apparent Km value of acetylcholinesterase from the field strain was 56% of that from the susceptible strain. By comparing the predicted amino acid sequences of a *S. frugiperda* ace-1 fragment from a chlorpyrifos susceptible strain with the corresponding sequence isolated from an 18.1-fold resistant strain from Brazil, Carvalho et al., (2013) were then able to identify the three previously described point mutations (Figure 5) [78].

Genotyping revealed that of the three, the A201S allele was present at relatively low frequency (17.5%) while G227A and F290V were present at higher frequency (67.5% and 32.5%, respectively) [78]. More recently, Boaventura et al., (2020) searched for the presence of these mutations in 34 different resistant populations of *S. frugiperda* collected from four different continents [79] and showed that F290V was the most frequent substitution in all populations tested. Similar results were obtained by Guan et al., (2020) on *S. frugiperda* individuals collected from China and Africa, except that only two positions (A201S, F290V) were found, with the F290V allele at higher frequencies [80].

## 3. Phase II: Conjugation

Following oxido-reduction and hydrolysis reactions performed by phase I enzymes, xenobiotic metabolites (including insecticides) are then conjugated to small hydrophilic molecules by phase II enzymes, a group of transferases that metabolize hydrophobic compounds containing nucleophilic or electrophilic groups [81].

### 3.1. Glutathione-S-Transferases (GSTs)

Glutathione-S-transferases (GSTs, EC 2.5.1.18) are one of the most important classes of this group whose biotransformation reaction leads to the generation of hydrophilic metabolites, which are readily excreted by efflux transporters such as ABC transporters. In arthropods, GSTs are highly diverse and are involved in a variety of cellular functions, from the detoxification of a wide range of both endogenous and xenobiotic compounds, to intracellular transport, hormone biosynthesis and reduction of oxidative stress [82]. GSTs primarily catalyze the conjugation of electrophilic lipophilic compounds with the thiol group of reduced glutathione (GSH) but are also capable of catalyzing a dehydrochlorination reaction using reduced glutathione as a cofactor. The enzymatic structure of cytosolic GSTs classically consists of hetero- or homo-dimeric proteins, with each monomer consisting of a highly conserved amino-terminal domain providing the GSH-binding site (G-site) while the carboxyl terminal domain interacts with the hydrophobic substrate (H-site) [82].

In insects, cytosolic GSTs belong to a diverse gene family divided into six classes (Delta, Epsilon, Omega, Sigma, Theta, and Zeta) based on their substrate specificities and phylogenetic relationships [83]. The growing number of available genomes reveals a large disparity in the total number of GSTs among insect species, with diversity ranging from low in hymenopterans (eight GSTs in *Apis mellifera*) to high in dipterans (39 in *Culex quinquefasciatus*) [84]. This variability is mainly due to genetic expansions observed in the Insecta-specific Delta and Epsilon classes due to multiple duplication events [85].

Insect GSTs have been intensively studied for their role in insecticide resistance: reports correlating high levels of GST activity with high resistance to various pesticides, including organophosphates, organochlorines, cyclodienes, and pyrethroids, exist for many species and among them *S. littoralis* and *S. frugiperda* [86]. This phenomenon relies on the precise regulation or induction of GST expression by xenobiotic compounds, and in particular the Epsilon class (GSTe) [87,88]. Here, we will focus on spodopteran GSTes that have been shown to contribute to insecticide response and resistance.

#### 3.1.1. Phylogeny of GST Epsilon

**Figure 6:**
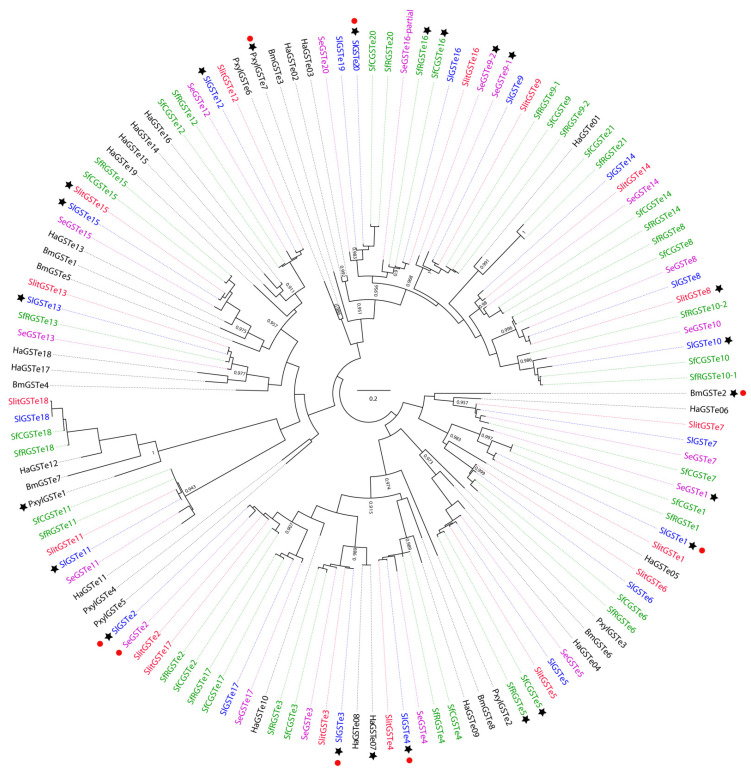
Maximum-likelihood phylogeny of lepidopteran epsilon glutathione-S-transferases (GSTes). The tree was built from amino-acid sequences of GST repertoires of *S. littoralis* (branches colored in red), *S. frugiperda* Corn and Rice strains (green), *S. litura* (blue), *S. exigua* (purple), *H. armigera, P. xylostella* and *B. mori* (black). Likelihood-ratio test values are only indicated in the internal nodes defining orthogroups, when aLRT > 0.9. Black stars indicate that GSTes are overexpressed in insecticide-resistant strains or when exposed to pesticides. Red dots indicate GSTes with demonstrated in vitro/in vivo insecticide resistance. The scale bar represents 0.2 expected amino-acid substitutions per site.

GST-mediated resistance could be triggered by different mechanisms, including gene amplification by multiple duplication events could lead to enhanced detoxification of insecticides underlying of the resistance process. Based on the recent availability of chromosome-level assembly genomes from spodopterans, we re-annotated GST epsilon class. For convenience, we reconciled the previous nomenclature with our new annotation to generate a unified dataset (see Supplementary Materials Table S2) [1,28]. Overall, GSTe accounts for nearly half of the total number of GSTs identified in the Spodopteran genomes (20 over 40 on average). Our phylogenetic analysis (Figure 6) revealed clear orthologous relationships among the four species studied with 1:1 orthologs in almost all cases, and with epsilon GSTs of *H. armigera,* suggesting that this great diversification is occurring in the Noctuoidea clade, and especially in highly polyphagous species.

**Figure 7:**
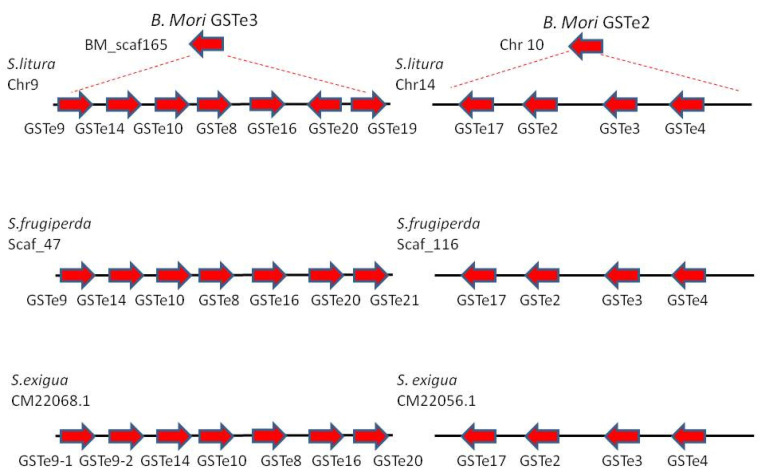
GSTe clusters derived from *B. mori* GSTe2 and GSTe3 identified in the genomes of *S. litura, S. frugiperda* corn and rice strains and *S. exigua* (adapted from [1]).

Such diversity could be explained by tandem and segmental gene duplications as demonstrated by the phylogenetic branching that corresponds to the genomic clustering of GSTe (Figure 6). Furthermore, synteny studies support this diversification, with half of the GSTe derived from only two duplication events, arguing that these clades share a common GST ancestor (in an ancient Lepidoptera ancestor) even though their genetic expansions occurred independently (Figure 7, [1]).

This extensive genetic amplification does not directly support a demonstration of insecticide resistance per se; however, this highly diverse class is expected to have ancient roles unique to polyphagous lepidopterans, such as the removal of harmful chemical compounds from natural sources. According to the pre-adaptation hypothesis, phytophagous insect species with more diverse diets are likely to acquire resistance to more diverse insecticides [89], and thus genetic diversity may underlie the origin and evolution of insecticide resistance.

#### 3.1.2. GST Activity against Insecticides

The biochemical mechanisms of GST-based insecticide resistance are classically associated with the conjugation of GSH to the pre-inactivated phase I compound. Such conjugation resistance has been demonstrated for different classes of organophosphate or pyrethroid insecticides [48,82]. Moreover, dehydrochlorination reactions using GSH as a cofactor have been implicated in resistance to the organochlorine DDT [6] making GSTs potential phase I enzymes for hydrochloric compounds. In addition to these direct modes of action, GST has also been shown to reduce lipid hydroperoxides produced as a result of insecticide-induced damages [90]. This peroxidase activity of GSTs protects tissues from an excess of reactive oxygen species (ROS), which can be more harmful than the pesticide itself [91]. Finally, GSTs are also capable of simple binding and sequestration activity towards various compounds, such as pyrethroids [86].

In spodopterans, most in vitro insecticide studies rely on the use of competitive 1-chloro-2,4-dinitrobenzene (CDNB) assays, a method that compares the amount of CDNB, the universal substrate of GST, in the presence or absence of a competitor, i.e., an insecticide. While this technique clearly indicates an interaction between a GST and a pesticide, it does not decipher the mechanism by which this interaction occurs. *S. litura* SlGSTe2 and SlGSTe3 were the first spodotperan GSTes to be characterized in this manner, showing differential activity toward DDT and deltamethrin between the two enzymes, with SlGSTe2 having the greater activity toward the insecticide [92]. The SeGSTe2 ortholog was further characterized showing activity toward metaflumizone, indoxacarb, monosultap, chlorpyrifos, malathion, cyhalothrin, and imidacloprid, suggesting a conserved insecticide metabolizing function [93]. Similarly, GSTe1 was carefully analyzed in *S. litura* and *S. exigua* species [94,95]. CDNB competition assays showed activity towards chlopyrifos, malathion, phoxim, deltamethrin and cypermethrin and the specific activities of GSTE1 were further analyzed using high-performance liquid chromatography (HPLC), confirming their active binding to chlorpyrifos and cypermethrin. Interestingly, GSTe1 is also active towards secondary plant metabolites [96] and has strong peroxidase activity. Therefore, this enzyme has multi-detoxification properties with a wide range of substrates, potentially conferring insecticide resistance.

In vivo studies have also confirmed the direct involvement of GSTs in insecticide tolerance. In Cheng et al., (2019), injection of siRNAi against SlGSTe20 and SlGSTe3 increased the sensitivity of *S. litura* larvae to imidacloprid, and the recombinant proteins subsequently showed activity towards diazinon, permethrin, chlorfenapyr, and bendiocarb [97].

#### 3.1.3. Resistance through Over-Expression of GST Epsilon

GST overexpression has often been a prerequisite for the identification of enzymes potentially involved in insecticide resistance [48]. Thus, many studies have identified candidate GSTes whose expression could be altered upon exposure to insecticides. In *S. litura*, SlGSTe1, e2, e3, e4, e10, e11, e12, e13, e15 and e20 showed overexpression following exposure to various pesticides such as tebufenozide, carbaryl, DDT, malathion, deltamethrin, chlorpyrifos and imidacloprid [1,92,98,99]. It is noteworthy that among these 10 over-expressed genes, no specific phylogeny-related pattern could be assigned (Figure 6) indicating that, rather than defining an insecticide-specific clade, the induced GSTe genes are diverse with potentially redundant and/or complementary activities. Similar patterns are observed in *S. littoralis* and *S. exigua* when exposed to deltamethrin or lambda-cyhalothrin, chlorpyrifos and chlorantraniliprole, respectively [94,100]. Nevertheless, induction after exposure may be only part of the animal’s response to a given toxicant and does not necessarily imply involvement in resistance [90]. Therefore, in an effort to find genes directly associated with insecticide catabolism, studies focusing on the expression profiles of genes highly expressed in resistant strains compared to the susceptible population have indicated potential candidates in GSTe as directly responsible for insecticide resistance. In *S. frugiperda*, SfGSTe5 was associated with an OP-resistant strain using a transcriptomic approach [78]. SfGSTe5 is the ortholog of *Plutella xylostella* PxylGSTe3, an epsilon GST induced and capable of metabolizing the organophosphate insecticides parathion and methylparathion [101,102]. In an RNAseq experiment comparing indoxacarb-resistant and susceptible *S. litura* populations [103], SlGSTe4 and SlGSTe20 were the only GSTs overexpressed. Interestingly, both of these GSTes are active against imidacloprid, diazinon, permethrin, chlorfenapyr, or bendiocarb [97,101], indicating potential cross-activity of SlGSTe for various insecticides. This complex pattern of gene expression regulation must be orchestrated by specific regulatory operators that can effectively govern down-or overexpression upon exposure but could also be affected by mutations in resistant strains to account for specific activations. Promoter sequence analysis revealed that some *S. litura* GSTs harbor the same cap ‘n’ collar ‘C/muscle aponeurosis fibromatosis (CncC/Maf) binding site and the aryl hydrocarbon receptor/nuclear aryl hydrocarbon receptor translocator (AhR/ARNT) binding site [84,94]. These transcription factors coordinately regulate GST expression through intracellular production of ROS induced by insecticide exposure. In a CncC RNAi experiment, Shi et al., (2020) demonstrate that this transcription factor not only enhances indoxacarb sensitivity in susceptible and resistant strains of *S. litura*, but is also involved in the down-regulation of detoxification genes related to indoxacarb resistance, confirming its central role in insecticide-resistance mechanisms [104].

#### 3.1.4. Resistance through Point Mutations in GST Epsilon

**Figure 8:**
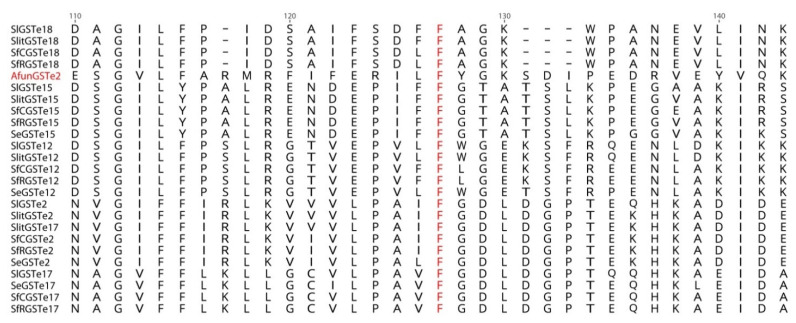
Alignment of a subset of GSTes from *S. litura* (Sl) *S. frugiperda* corn and rice strains (SfC and SfR), *S. exigua* (Se), *S. littoralis* (Slit) and AfunGSTe2 from *Anopheles funestus* (Afun, genbank accession number KC800363.1) harboring a L119F substitution. The L119F mutation (in red) is associated with DDT resistance in Anophelines [6].

Insecticide resistance by GSTe has been clearly established in *Anopheles* where a key residue, Phe120, has been implicated in the increased binding of DDT to GSTe2 [6]. Such a substitution is also observed in 5 GSTes from spodopteran species (GSTe2, e12, e15, e17, e18, Figure 8) indicating potential conservation of resistance processes between these species. Thus, further investigations are needed to fully understand the detailed mechanisms of pesticide-GSTes interaction and their precise contribution to insecticide resistance.

## 4. Phase III: Elimination/Export

The last phase concerns the elimination or excretion of metabolites outside the cell. Metabolites have been rendered less toxic by the reactions they underwent in phase I and/or phase II but they can also be directly excreted without prior chemical modification. This phase mainly involves ATP-binding cassette transporters.

### 4.1. ATP-Binding Cassette Transporters (ABCs)

ATP-binding cassette (ABC) transporters are proteins that use the energy of ATP hydrolysis to transport substrates such as amino acids, lipids, peptides, sugars and drugs across cell membranes. ABC transporters are classified into eight subfamilies from letter A to H based on similarities in their ATP binding domain. Full transporters (FT) consist of two cytosolic nucleotide-binding domains (NBDs) and two transmembrane domains (TMDs) whereas half transporters (HT) have only one of each domain and must dimerize to form a functional transporter. The NBD is involved in ATP binding and hydrolysis. The TMD consists of 5-6 transmembrane segments and is responsible for substrate specificity. In insects, their number varies from 32 in *Nilaparvata lugens* [105] to 82 in *Plutella xylostella* [106] while in the available genomes of Noctuidae about 50 ABCs have been found so far. Some subfamilies are conserved well from humans to arthropods. For example, ABCD, ABCE, ABCF have clear orthology and similar suspected physiological roles such as the transport of acyl-CoA molecules into the peroxisome, ribosome biogenesis and translation. Moreover, some ABC transporters subfamilies are involved in chemotherapy resistance in humans and pesticides resistance in insects. Their names reflect these roles, the B subfamily is known as multidrug-resistance protein (MDR) or P-glycoprotein (P-gp) while the C subfamily is known as multidrug-resistance associated protein (MRP). For example, the human MDR1 (ABCB1) excludes chemotherapeutic agents and confers resistance in several types of cancer when over-expressed [107]. MDR1 orthologs in *Drosophila Mdr50* and *Mdr65* are over-expressed in DDT-resistant strain 91-R. Knockdown of each gene in this strain by RNAi increases the susceptibility of flies to DDT [108]. These examples, in humans and *Drosophila,* illustrate one of the mechanisms of resistance involving ABC transporters: over-expression of the transporter which allows exclusion of a greater amount of xenobiotics. An additional mechanism in the case of insecticide resistance can be mentioned and corresponds to the mutation of the target. Indeed, ABCs are one of the receptor proteins of *Bacillus thuringiensis* (Bt) toxins as are cadherin-like, aminopeptidase-N or alkaline phosphatase, and a modification in the sequence of these receptors can confer resistance (for a recent review see [109]).

#### 4.1.1. Resistance through Point Mutations in ABCs, the Case of ABCC2

**Figure 9:**
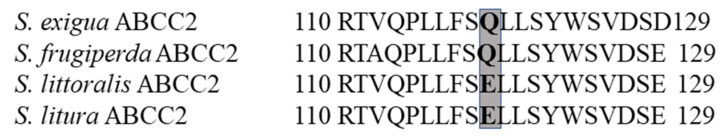
Alignment of a 20 amino acid sequence of ABCC2 from four *Spodoptera* species, presence of a glutamine or glutamic acid at position 119. Genbank accession number, *S. exigua* AIB06822, *S. frugiperda* AUO38740, *S. littoralis*, *S. litura* XM_022967434.

ABCC2 was first identified as a receptor for one of the Bt crystal toxins (Cry), Cry1Ac in the lepidopteran *Heliothis virescens* [110]. In this study, a strain of *H. virescens* was selected in the laboratory for several years with Cry1Ab and Cry1Ac toxins. Very high levels of resistance were obtained as well as a loss of membrane binding for the toxins. A deletion in exon 2 of *ABCC2* resulting in a 99 amino acid protein truncation was identified as the cause of resistance to Cry1Ac [110]. This study paved the way to investigating the role of ABC transporters in resistance to Bt-toxin. Special attention is given to the four species of interest in this review. Annotations of ABC transporters in the genome of *S. litura*, *S. frugiperda*, *S. exigua* and in the transcriptomic data of *S. littoralis* were mainly performed automatically and only a few subfamilies were annotated manually by experts (G. Le Goff, personal communication). In a recent publication, Liu et al., (2018) showed that the sensitivity towards Cry1Ac toxin differed by a factor of 65 between the more tolerant *S. litura*, and *S. frugiperda* [111]. SlABCC2 and SfABCC2 share 97% identity. Fragment substitutions and point mutations in the transporter expressed in insect cells allowed the authors to identify the amino acid at position 125 as the key to this difference in sensitivity. A glutamine is present at this position in *S. frugiperda* while it is a glutamic acid in *S. litura*. By aligning the sequences of ABCC2 between the four *Spodoptera* species (*S. exigua, S. frugiperda, S. littoralis* and *S. litura*), it is possible to predict their susceptibility to Cry1Ac toxin. *S. exigua* should be susceptible like *S. frugiperda* while *S. littoralis* should be more tolerant like *S. litura* (Figure 9).

Three-dimensional structure predictions suggest that the G or E at position 125 is localized in the extracellular loop 1 (ECL1). Liu et al., hypothesize that it may have a role in toxin binding, which would explain these differences in sensitivity [111]. The importance of ECL1 in Bt toxin selectivity was previously demonstrated in *B. mori*, where replacement of certain amino acids in the ECL1 loop between BmABCC2 and BmABCC3 resulted in increased binding affinity of Cry1A toxins [112]. These variations in toxin receptor sequence may also explain the spectrum of specificity of Bt toxins. Indeed, some toxins primarily target lepidopterans, for example Cry1 toxins, while others are specific to Coleoptera, such as Cry3 [113].

**Figure 10:**
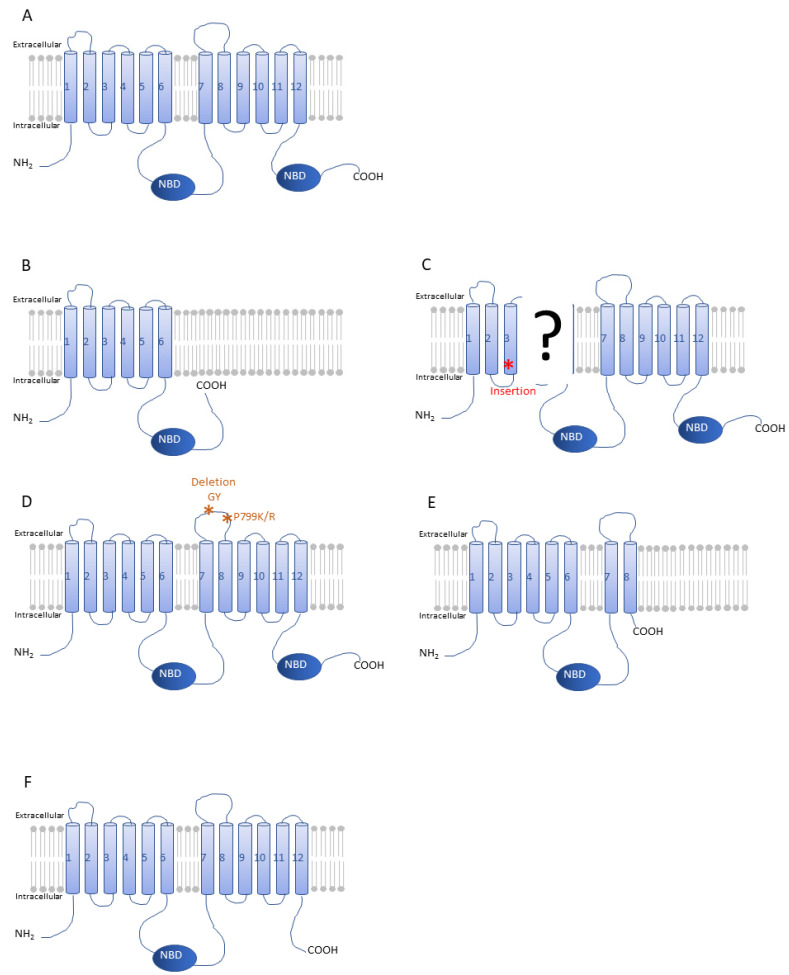
Protein structure of ABCC2 of *Spodoptera* spp from susceptible or resistant populations. (**A**) Protein structure of SfABCC2 and SeABCC2 wild-type. (**B**,**C**) Protein structures of SfABCC2 in resistant populations from Puerto Rico, * corresponds to an insertion of amino acids [114,115]. (**D**,**E**) Protein structure of SfABCC2 in resistant populations from Brazil [80,116]. (**F**) Protein structure of SeABCC2 from a laboratory selected strain [117].

Larger changes in the sequence of ABCC2 (Figure 10A) have been associated with resistance in some of these *Spodoptera* spp., especially in field-resistant *S. frugiperda* populations. Indeed, resistance to transgenic maize expressing the Cry1Fa toxin has been reported since 2007 in Puerto Rico for *S. frugiperda.* Banerjee and colleagues have demonstrated that the resistance was due to mutations in the SfABCC2 toxin receptor [114]. These mutations result in a truncated protein that loses the second transmembrane domain and at the same time toxin binding. A nine-base deletion (position 39–47) and a two-base insertion (GC at position 2218) lead to a frameshift and the occurrence of a premature stop codon. The truncated protein is 746 amino acids (Figure 10B) whereas the ABCC2 of the susceptible strain encodes a protein of 1349 amino acids (Figure 10A). The frequency of mutated *SfABCC2* in Puerto Rico increased between 2007 and 2009 from 1% to 42% and stabilized at 55% in 2017. This truncated receptor was not found by the authors in the populations from Florida or the Dominican Republic [114].

Another group confirmed these results. Flagel et al., isolated a population of *S. frugiperda* from a site in Puerto Rico in 2010 and this population was selected in the laboratory with Cry1Fa for 50 generations [115]. The resulting strain had a resistance factor of 500 for Cry1Fa and 87-fold cross-resistance to Cry1A.105 compared to the susceptible reference strain. They found the same GC insertion in the ABCC2 sequence that creates a stop codon at position 747 and results in a truncated protein. By conducting a sampling campaign in various localities of Puerto Rico and Brazil, the mutation was to be found in Puerto Rico only. In addition to this allele, the authors mention the existence of a second resistance allele and the insertion of a sequence in the fourth exon of the gene. However, sequencing difficulties due to repeated sequences did not allow them to identify precisely the effect of this insertion on the *ABCC2* sequence (Figure 10C) although an aberrant splicing of this allele 2 was suspected.

Furthermore, a recent study reported resistance to Cry1F toxin in isolated Brazilian populations of *S. frugiperda* [116]. The authors identified two mutations in the extracellular loop 4 (ECL4) of ABCC2: a deletion of two amino acids (GY) at positions 788 and 789 and the change of a proline to either lysine or arginine at position 799 (Figure 10D). Unlike the resistance reported in Puerto Rico, these mutations do not cause premature termination of the sequence. Expression of mutated ABCC2 in insect cells confirmed the role of these mutations in toxin binding. Analysis of populations collected in different regions of Brazil showed a high frequency of the GY deletion as well as many rare alleles that result in sequence changes between amino acids 783–799. According to the authors, these results reinforce the primary role of the ECL4 extracellular loop in Cry1F toxicity. Further analysis is needed to demonstrate the role that these mutations may have in resistance to other toxins. Indeed, in addition to resistance to Cry1F other studies on Puerto Rican populations with truncated SfABCC2 have shown cross-resistance for Cry1A.105, Cry1Ac, and Cry1Ab toxins [114,115]. Do mutations in the ECL4 loop also confer these cross-resistances? Another mutation in ABCC2 has been identified in Brazilian populations corresponding to the insertion of 12 bases at the intracellular loop between transmembrane domains 8 and 9 resulting in a premature stop codon [80]. The truncated protein lost the last four transmembrane segments and the second intracellular ATP-binding domain (Figure 10E). The study was performed on insects collected three years after those of Boaventura et al., (2020) and changes in the use of transgenic crops may have occurred. The authors did not investigate the effect of this mutation on resistance and of Bt toxin binding. Instead, they tested whether invasive populations of *S. frugiperda* (also sampled in Africa and Asia) carried mutations known to cause insecticide resistance.

None of the mutations identified so far in ABCC2 were found in these invasive populations. This truncated protein retains an intact extracellular loop 4 and thus potentially to bind to the Cry1F toxin. Further experiments are needed to determine if it can confer resistance to some of the Bt toxins.

Looking at other *Spodoptera* species, resistance to some Bt toxins was reported as early as 1994–1995 for *S. littoralis* and *S. exigua* respectively [118,119]. However, in both cases, these were laboratory selected strains. No resistance has been reported to date for *S. litura* in the Arthropod Pesticide Resistance Database although one study mentioned the development of resistance to Cry1C and Vip3A toxins in a laboratory selected strain [120]. But these studies did not involve ABCC2. In *S. exigua*, a strain was selected with a commercial *B. thuringiensis* product, Xentari. The main toxins contained in this product are Cry1a and Cry1Ca. The selected strain (Xen-R) showed a resistance factor of more than 1000 [121]. Bulk segregant analysis based on high-throughput sequencing identified the region of the genome carrying the major resistance loci, which contained three genes encoding *ABCC1*, *ABCC2* and *ABCC3* [117]. ABCC2 and ABCC3 played a major role in resistance. ABCC2 contained a mutation in the resistant strain, which resulted in the loss of the ATP binding domain II caused by the truncation of 82 terminal amino acids (Figure 10F). However, the binding of Cry1Ca to brush border membrane vesicles was not significantly different between resistant and susceptible strains. RNAi experiments confirmed the role of ABCC2 and ABCC3 in the resistance to Cry1A and Cry1Ca in *S. exigua* [117]. In addition, Pinos and co-workers showed that the loss of the second nucleotide binding domain did not affect the binding of Cry1A toxins [122]. Indeed, expression of the truncated transporter in Sf21 insect cells conferred sensitivity to Cry1A, as specific binding of the toxin was still effective. These results suggest that this domain is not required for a functional toxin receptor [122]. Nevertheless, the use of techniques such as CRISPR/Cas9 validated the main role of ABCC2 as a receptor for Cry1Ac and Cry1Fa toxin in *S. exigua* [123]. The knock-out (KO) strain for SeABCC2 was more resistant than the parental strain by a factor of 470 and 240 for Cry1Ac and Cry1Fa, respectively. Using the same technique in *S. frugiperda* to produce a KO for ABCC2 conferred resistance to Cry1F toxin [124].

#### 4.1.2. Resistance through Over- or Reduced Expression of ABCs

Data on the involvement of ABC transporters in resistance to chemical insecticides in *Spodoptera* spp. are scarce, as it is in Lepidoptera in a broader sense. A few examples exist, such as the involvement of *H. armigera* Pgp1 insecticide transport. HaPgp1 expression was induced in the larval gut after exposure to abamectin [125]. When the expression of this transporter is suppressed by RNAi, larvae became more sensitive to the insecticide and mortality increased from 26% to 84% for a fixed abamectin dose of 0.4 µg/g diet [125]. Another study confirmed the role of HaPgp in abamectin transport, as the use of a specific Pgp inhibitor, verapamil, increased the sensitivity of larvae towards abamectin [126].

The development of the CRISPR/Cas9 technique has paved the way to study the role of ABCs in the transport of chemical insecticides and potentially in the development of resistance. Here, we report the few examples in which an increase in LD_50_ was observed for insecticides after KO of certain ABC transporters in *Spodoptera* spp. In *S. exigua*, the ability of ABCB1 to transport 12 insecticidal molecules from 10 different chemical families was investigated by knocking out this protein using CRISPR/Cas 9 [127]. The ABCB1 KO strain showed a 2.73-fold and 3.01-fold increase in sensitivity to abamectin and emamectin benzoate, respectively, while no significant difference was observed for the other insecticides tested. In *S. frugiperda*, the KO of ABCC2 rendered the insects 7.8 and 3.1 times more sensitive to abamectin and spinosad, respectively. Similarly, a reduction in tolerance to these two insecticides was observed for the ABCC3 KO by factors of 4.5 and 2 [128]. Another study reported that SfABCC2 KO does not induce resistance to insecticide molecules such as bifenthrin, chlorantraniliprole, spinetoram and acephate [124]. To our knowledge, there are no CRISPR/Cas9-mediated ABC KO data for *S. litura* and *S. littoralis* to date.

#### 4.1.3. Resistance and Regulation of ABCs Expression

While the mechanisms leading to the acquisition of chemotherapy resistance through changes in ABC transporters expression of are well known in humans, those leading to pesticide resistance are still largely unexplored in insects. For example, over-expression of MDR1 (ABCB1) in human tumors has been associated with increased gene copy number through chromosomal region amplification, epigenetic modifications and single nucleotide polymorphisms (SNPs) [129]. In insects, the acquisition of resistance may involve either under- or over-expression of a given ABC transporter. Indeed, in the case of Bt toxins, decreased expression of the ABC receptor can confer resistance, while conversely, an over-expression of ABCs that can transport insecticides would allow the development of resistance. MicroRNAs (miRNA), which are small non-coding RNA sequences (between 19 and 24 nucleotides in length) are known to regulate gene expression. The miRNA miR-998-3p has target sites in the ABCC2 coding sequence (CDS) in several Lepidoptera species [130]. The involvement of these sites in the regulation of ABCC2 has been demonstrated in *P. xylostella*. MiR-998-3p was over-expressed by a factor of two in a Cry1Ac-resistant strain (GX-R) compared to a susceptible strain and ABCC2 expression in the larval midgut was reduced by approximately 50%, demonstrating the involvement of this miRNA in Bt resistance. A conserved target site for miR998-3p had been found in the four *Spodoptera* spp. as well as one and two non-conserved sites for *S. exigua* and the other three species respectively ([130] and the present study). MiR-998-3p could play a role in the acquisition of resistance in these species but to the best of our knowledge there has been no study has so far reported such cases and more generally the role of miRNAs in insecticide resistance remains largely unexplored.

Another regulator of *ABCC2* expression is the transcription factor Forkhead box protein A (FoxA). Its transfection into Sf9 cells (*S. frugiperda* cell model) induces the expression of ABCC2 and ABCC3 and results in increased susceptibility of the cells to Cry1Ac toxin [131]. Potential resistance could arise through under-expression of this transcription factor but has not been described at this time.

Cap ‘n’ collar isoform C (CncC) is a major transcription factor for controlling the expression of detoxification genes in insects [12]. CncC is constitutively overexpressed in a Chinese field population of *S. exigua* resistant to chlorpyrifos and cypermethrin [94]. Several studies on this resistant strain showed that CncC is involved in controlling the expression of detoxification genes including P450s (*CYP321A8*, *CYP321A16*, and *CYP332A1*) [11,132] and GSTs (*GSTo2*, *GSTe6*, *GSTd3*) [94]. However, none of these studies mention the role of CncC in the expression of ABC transporters. Nevertheless, this role is not excluded and an RNAseq analysis would allow a global approach to identify CncC-regulated genes in this resistant population. In *Drosophila*, a DDT-resistant strain (91R) constitutively overexpressing CncC exhibits multifactorial resistance involving some P450 and GST in addition to ABC transporters [108]. None of these studies investigated the underlying cause of CncC overexpression. In other *Spodoptera* species, CncC regulates the expression of several detoxification genes involved in indoxacarb resistance, including the ABC transporter SlituABCH-1 in *S. litura* [104]. Although the CncC gene from *S. frugiperda* has been cloned [133], it has not been shown to control of the expression of resistance-related ABC transporters and, to date, this information is also lacking in *S. littoralis.*

A number of questions remain unanswered. What is the mechanism behind the over-expression of CncC in some resistant populations? What other regulatory mechanisms could control the expression of ABC transporters involved in resistance? Do epigenetic regulations play a major role in the expression of certain ABC transporters and the acquisition of resistance, as has been observed in humans?

## Figures and Tables

**Table 1:** Assessment of insecticide resistance worldwide for the four species of *Spodoptera*, data extracted from the Arthropod Pesticide Resistance Database (http://www.pesticideresistance.org, accessed on 18 May 2021).

Insecticide Chemical Class	*S. exigua*	*S. frugiperda*	*S. littoralis*	*S. litura*
Avermectins	Abamectin (#31), emamectin benzoate (#48)			Abamectin (#43), emamectin benzoate (#27)
Benzoylurea	Chlorfluazuron (#9), diflubenzuron, lufenuron (#10), teflubenzuron	Lufenuron (#2), triflumuron	Diflubenzuron, teflubenzuron (#3)	Lufenuron (#8)
*Bacillus thuringensis*	Bt var. unspecified (#10), var aizawai, var. kurstaki		Bt var unspecified (#3), var aizawai	
Bt toxins	Cry1Ca	Cry1Aa, Cry1A.105, Cry1Ab (#2), Cry1Ac (#3), Cry1F (#54), Cry2Ab2 (#2), Vip3A (#3)		
Carbamates	Methomyl (#18), thiodicarb	Carbaryl (#7), methomyl (#6), thiodicarb (#2)	Carbaryl (#2), methomyl (#2),	Carbaryl (#2), methomyl (#38), thiodicarb (#33)
Cyclodienes	BHC/cyclodiene—unspecified in literature (#3), endosulfan (#19)	Aldrin, dieldrin, lindane (#2)	Endrin, toxaphene (#2)	Endosulfan (#31), lindane
Diamides	Chlorantraniliprole (#26), cyantraniliprole, flubendiamide (#5)	Chlorantraniliprole (#2), flubendiamide (#2)		Chlorantraniliprole (#11)
Diacylhydrazines	Methoxyfenozide (#25), tebufenozide (#19)		Tebufenozide	Methoxyfenozide (#36), tebufenozide
Neonicotinoids				Acetamiprid
Organochlorine	DDT (#4)	DDT (#3)	DDT	DDT (#2)
Organophosphates	Chlorpyrifos (#48), parathion-methyl (#3), profenofos (#22), quinalphos (#9)	Acephate, chlorpurifos (#7), diazinon (#2), dichlorvos, malathion (#2), parathion-methyl (#4), sulprofos, trichlorfon,	Acephate, azinphos-methyl, chlorpyrifos (#4), fenitrothion, leptophos, methamidophos, methidathion, monocrotophos (#4), parathion (#2), parathion-methyl (#3), profenofos, sulprofos, triazophos, trichlorfon	Chlorfenvinphos, chlorpyrifos (#55), diazinon, dichlorvos, malathion, monocrotophos (#5), phoxim (#4), profenofos (#56), quinalphos (#9), triazophos (#2), trichlorfon
Oxadiazines	Indoxacarb (#44), metaflumizone (#4)		Indoxacarb	Indoxacarb (#50), metaflumizone
Phenylpyrazoles				Fipronil (#15)
Pyrethroids	Bifenthrine (#13), cyfluthrin (#2), cyhalothrin-lambda (#2), cypermethrin (#56), cypermethrin-beta, deltamethrin (#43), fenpropathrin (#14), fenvalerate (#5), permethrin (#2), pyrethroids-unspecified in literature (#3)	Bifenthrine, cyfluthrin, cyhalothrin, cyhalothrin-lambda (#11), cypermethrin (#2), cypermethrin-zeta, deltamethrin (#2), fenvalerate, fluvalinate, permethrin (#5), tau-fluvalinate, tralomethrin	Cypermethrin (#4), deltamethrin (#2), fenvalerate, flucythrinate, permethrin	Bifenthrine (#33), cyfluthrin (#12), cyfluthrin-beta (#9), cyhalothrin (#3), cyhalothrin-lambda (#11), cypermethrin (#49), cypermethrin-beta (#15), deltamethrin (#50), esfenvalerate (#9), fenpropathrin (#10), fenvalerate (#7), pyrethrins
Pyrroles	Chlorfenapyr (#11)			Chlorfenapyr (#5)
Spinosyns	Spinetoram, spinosad (#56)		Spinetoram (#2), spinosad (#2)	Spinosad (#39)

# number of cases reported in Arthropod Pesticide Resistance database.

## Supplementary Materials

The following are available online at https://www.mdpi.com/article/10.3390/insects12060544/s1, Table S1: Protein sequences of *S. littoralis* carboxyl/cholinesterases, Table S2: Spodopteran GSTes reconciled nomenclature, with new annotations combined with previous studies.
