# Supplementary material for: Resistance in the Genus Spodoptera: Key Insect Detoxification Genes
Source: Insects. 2021 Jun 11;12(6):544. doi: 10.3390/insects12060544 (PMC8230579; doi:10.3390/insects12060544)
Supplement: Supplementary file 1 [file insects-12-00544-s001.zip › insects-1245927-supplementary.pdf]

Table S1: CCE transcripts of *S. littoralis*

CLADE	Sequence name (RNAseq)	sequence (aa)	aa length
clade016	**SlitCXE67**	MRLITTVGVLILCVIHNISCDDDVTLVEVEQGQLRGQVLDAVVGDFQYLSYKGIPYAKPPVGKLRFKDPEYLDSWE<br>GVLNATEHGSVCPQFNPITAVYTSGSEDCLFLNVYTRNLTPDAPRPVVVFIHGGAYIFGSGNTDLYRPDYLLANDV<br>IVVTINYRLGVLGFLSLGNEDVPGNAALKDQVTALKWVQNNIEKFGGNPNSVTIVGDTAGGASVTLHMLSPMSKGL<br>FHKAVAMSGSATCDFGLTYKHVEKAKIFGSALKCENVEDSAALLDCLQAADYNAFYSITPTVLASEEITDVLFKME<br>HFTPVIEKKTGNNFLTEDYYSALTKGHVNKDVDFIIGYSSQEAVLLIDLFNASYVDLYSRYKELFTPSEILIKSTP<br>DTNLNVAKSVKEFYFGEKPVKTENLNLFVKYSSSASIGYHAQRYANKWANFGKKTYFYIFDGFTDWNFFGQQGVKY<br>GIEKASHFDITYYLFYPESLNWSIDTDSVEYQVTKQLTTSLTNFAKSSDPSVDGVTWPQYSESSKAYVTFGNDGPV<br>VGYGPDEDDYNFWAGVYKSAGIPF	556
	**SlitCXE54**	MMSTVLRIALLCVVLSEALAMVQVRVSDGLLEGEVVHNEYGGTYFSFLGIPYAQPPLGDLRFKAPQPPKPWDGVRS<br>AKEFGPKCFQYDLFVDKGHVTGSEDCLYLNVYTPDVTPVKPLPVMVWIHGGGLVSGAGDDQEYGPKFLVRQDVILV<br>TLNYRLEVLGFLCLDTEDAPGNAGMKDQVAALRWVKENIANFGGDPDNVTIFGESAGGVSVTYHLMSPMSKGLFQR<br>AIAQSGVSVSYWAQAYKPRERGFALARKLGFYSDDVKDVYEFLKQQPIENLIKTKVPITYSEKERTNVEVYFSVVE<br>EKQFGDNERFFYGDMIDAVSNGIHEGVDIMTGYTADEGIMGVAIFGDYKESLEQAKNFPQFFVSYPMSLSLSTNDQ<br>LELGNRLKEYYFKEQIRVPDHWESLKDFYSMDIFVFPTMRWIKLSARSKKNKIYLYKFTCYTELNIVSELMGVGHL<br>VGNKPVVAHSDDLMYLFSARNQTKLDMNSEEFKQIEVVTKLWTNFAKYGNPTPDDSFGVAWAPYSLENQDYLDIGN<br>ELKAGQAPDDEEIKFWENLLTEYGQKLY	560
	**SlitCXE55**	MVQVQVNEGWLEGEVVQNEYGGSYYSFKGIPYAQPPLGELRFKAPQPPKPWKDVRSAKQFGSKCYQYDAFFEKGKV<br>CGSEDCLYLNVYTPSIKPDKPLPVMFWIHGGSFISGAGDDDVYGPKFLVRQDVILVTFNYRLEVLGFLCLDTEDVP<br>GNAGMKDQVAALRWVNKNIANFGGDPNNITIFGESAGGASVTYHLISPMSKGLFRRAIAQSGTNVGYWAQAYKPRE<br>RGFALARKLGFYSDDPKQVYEFLKEQPLEALIKAAVPITYSEKARTYTEFYFSVAAEKQFGNSKQFFCGDVLDSVS<br>NGIHEGVDIMTGYTADEGILSLAIFGDISASLEQARNFPQLFISYPMSLTLSTNDQLELGKRIREYYFKNSISVPD<br>DWEKLEKFYAADLFVFPANRWIKLCAQSKKNKVYFYKFTCVSELNMISQMIGVGDLIKDKPVVAHADDLLYLFTPN<br>TSPIIDRNSKTFQHVEKVTKLWTNFAKFGNPTPDDSLGVTWTPYSVANKDYLDIGNELKPGHAPDDEEVQFWENVL<br>TEFNQKLY	540
	**SlitCXE56**	MVQVQVNEGWLEGEVVQNEYGGSYYSFKGIPYAQPPLGELRFKAPQPPKPWKDVRSAKQFGSKCYQYDAFFEKGKV<br>CGSEDCLYLNVYTPSIKPDKPLPVMFWIHGGGFISGAGDDDVYGPKFLVRQDVILVTFNYRLEVLGFLCLDTEDVP<br>GNAGMKDQVAALRWVNKNIANFGGDPNNVTIFGESVGGASVTYHLISPMSKGLFRRAIAQSGTNVGYWAQAYKPRE<br>RGFALARKLGFYSDDPKQVYEFLKEQPLEALIKAAVPITYSEKARTYTELYFSVAAEKQFGNNERFFCGDVLDSVS<br>NGIHEGVDIMTGYTADEGILSLAVFGDVSTSLEQARNFPQFFISYPMSLTLSTNDQLELGKRIREYYFKNSISVPD<br>DWEKLEKFYAADLFVFPANRWIKLCARSKKNKTYFYKFTCVSELNMISQTMGVGDLIKDKPVVAHADDLLYLFSPN<br>SSPIIDRNSKTFQHVEKVTKLWTNFAKFGNPTPDDSLGVTWTPYSVANKDYLDIGNELKPGHAPDDEEVQFWENVL<br>TEFNQKLY	540
	**SlitCXE57**	MVQVQVNEGWLEGEVVQNEYGGSYYSFKGIPYAQPPLGELRFKAPQPPKPWKDVRSAKQFGSKCYQYDAFFEKGKV<br>CGSEDCLYLNVYTPSSKPDKPLPVMFWIHGGGFISGAGDDDVYGPKFLVRQDVILVTFNYRLEVLGFLCLDTEDVP<br>GNAGMKDQVAALRWVNKNIANFGGDPNNITIFVGESAGGASVTYHLISPMSKGLFRRAIAQSGSNVGYWAQAYKPR<br>ERGFALARKLGFHSDDPKQVYEFLKEQPVEALIKAAVPITYSEKVRDNEIYFNVTAEKQFGNNEQFFYGDVLDSVA<br>NGTHEGVDIMTGYTADEGIMSLAVFGDISANLEQARNFPQFFITYPMSLTLSTNDQLELGKRIREYYFKNSISIPD<br>DWEKLEKFYAACQFLFPANRWIKLVARSKKNKTYFYKFTCVSELNMISQMMGVGDLIKDKPVVAHADDLLYLFSPN<br>TSPIIDRNSKTFQHVEKVTKLWTNFAKFGNPTPDDSLGVTWTPYSVANKDYLDIGNELKPGHAPDDEEVQFWENVL<br>TEFNQKLY	540
	**SlitCXE63**	MVQVRVNEGLLEGERVDNDYGGSFYSFKGIPYAQPPVGDLRFKAPLPPKSWDGVRSAKEFGPRSFQNDILMKTGLV<br>GDEDCLYLNVYTPEIKPDKPLPVMFWIHGGGFYCGSGNDDLYGPEFLIRHGVILVTINYRVDVLGFLCLDTEDIPG<br>NAGMKDQVQALRWVNKNIASFGGDPKNITIFGESAGGSSVSYHLISPMSKGLFKRAIAQSGVSTCAWAQAIEPRER<br>ALALARSLGCHSEDDKELYDFFKNQPKESLVGVQCPVATFEAFKGGLDIYFNVANEKKFGDNERFFYGDMLDVISN<br>SVHEGVEIMMGSTTEEGLISYADSEDIIKTLKLAKIYPEYFVNKLVAFSLPLKQQLKIGKEVRKFYFNDQINVPDD<br>WDKLINFTSMQNFVYPERQWAKHSAQGKKHKLYLYKFSCKTERNFIAKMRGMTEMLGNREVTCHADELPYLFNFKM<br>MPTKLDTASETFQLMERLMKLWTNFAKYGNPTPDDSLGAKWAPYTLENQEYLDIGNELKTGTAPDAEETQFWDKLY<br>EKYGL	537
	**SlitCXE64**	MVQVRVNEGLLEGEQVDNHYGGSFYSFKGIPYAQPPVGDLRFKAPLPPKPWDGVRTAKEFGPKSYQNDIFMNVGRV<br>GEEDCLYLNVYTPSIKPDKPLPVMFYIHGGGFFSGSGNDDLYGPELLVRHGVILVTINYRVDVLGFLCLDTEDIPG<br>NAGMKDQVQALRWVNKNIANFGGDPKNITIFGESAGGASVSYHLISPMSKGLFKRAIAQSGASTSPWAQAVEPRER<br>ALALARSLGFYSEDDKELYEFFKHQPMESIVGVPVSVATFEAFKGGIDVNFTVVNEKKFGENERFFYGDMIDAVSN<br>GVHEGVEIMTGTTTEEGLIAMGDLKDTKKALELARTYPEIFMNKYEAYNLPLKQQLKIGREIRKFYFNDQVNIPDD<br>WDKLMNFLSMQMFVYSTMQWVKYCVQAKKHKLYLYKFSCKSERNVMSFLRGQTEMLGSRRVTCHADELTYLFNPKL<br>MPKMDTASATFKMVERILTLWTNFAKYGNPTPDDSLGVNWAPYTLENQEYLDIGNELKAGTAPDAEETQFWDNLYE<br>KYEL	536
	**SlitCXE108**	MHTKISFFIFFTCFLQWIFCEYVEVEVAQGRLRGERLETVTGEAAYYSFKGIPYAKPPVGTLRFKDPLPPEPWDGV<br>RDATQHGSVCPQVELLNNIVIPGNEDCLFLNVYTPILTPETPLPVMVFIHGGGFKFGSGNVDVYGPDFLVAKDVIV<br>VTMNYRLDVLGFLALGTKEVPGNAGMKDQVLALRWVNENIENFGGDSRNITLFGESAGATAVAYHLVSPISKGMFQ<br>RAILQSGATSLDCFIPYKPRERAFVLGNSLNITTKNSTELLLSLQELPAVELLNRTAYLFASESITHIQFKFTPFT<br>PVIEEDFGQDMRFVSEDPFDSLDNGNVNDVDVMFSYNKHETLIMLPFYVNDNFRYIQRYNRYPELLVPSKILIKAN<br>TDDIYYLWNKINDFYFGDKSISVENMPEFIKYSSFASVVYDVHRFIRRWPSVGNVYLFKFESYSSRSYYGLQGASY<br>GIIGPAHFDDLFYVFDPKIMQFSLPINSTEYKMVQQMTTIFTNFAKYGNPTPDKSLGVFWPQYNRSKQAYAIIADN<br>ITIGYKPDAKDIKFWKEVLEYAHIKF	558

	**SlitCXE86**	MGDADCLPSGDPSARLPILHWISCEYVEVEVAQGQLRGELLDTVTGEAKYYSFKGIPYAKPPVGDLRFKDPQPPEP<br>WDGVRNATQHGSVCPQIDLLNSIVVPGSEDCLFLNVYTPNLTPENPLPVMVFIHGGGFKFGSGNVDVYGPDFLVAK<br>DVIVVTMNYRLDVLGFLALGTKEVPGNAGMKDQVLALRWVNENIESFGGDKQNITLFGESAGASAVGYHLVSPMSK<br>NLFQKAILQSGVPSMDPHIPYRTVERAFVLGNSLNITTKNSTELLLSLQELPAVELLNRTAFLFASESITHIVFKF<br>TPFTPIIEEDYGQEMIFVSEDPFDTLDSGNVSNVDIMFSYNKYEMLIMLPYYVNNTYRYIQRYNRYPELLVPSKIL<br>MKASSDDIYYLWNKINDFYFGNKSISVENMPEFIQYAGFASLVYDVHRFIRRWPQVGNVYLFKFECISSRNFYGLP<br>GAAYGLNGPSHFDDLFYVFDPKSLQFPLPTDSKEYKLVQQIATVFTNFAKYGNPTPDSTLGVSWPQYDKSKQAYAI<br>IADNITIDYKPDAKDIKFWRKVLEYAHIKF	562
clade001	**SlitCXE42**	MQWQTCVLLLCVTAILADDEWREVKTAQGPVRGRKHPTEDIYTFYNIPYATAPVGVDKFKAPLPPPVWLESYEAVD<br>EQVICPQPALPFQDLEIKNELPLVKQENCLIANIYVPNTNKKNLSVLVYVHGGGFVTGWGELFKATQLLKKKDIIV<br>VTFNYRLGVHGFLCLGTEDAPGNAGMKDQVALLRWVQKNIASFGGNPDDVTIIGYSAGSASVDLLMLSESTEGLFH<br>RVIPESGGNLAAFSIQRDPVEIAKTFARQLNFTNVDDIYALGEFYKTAPLELLTSDPFIDRTDSTLMFSPCVERNM<br>GEETFLTESPLTILKNGKYKKLPMLYGFANMEGLFRVNFFPLWKQKMNDKFSDFLPADLRFESEEEREDVANKVKR<br>FYFGDEPVSEVNIVEYVNFFTDVMFAHSMLWATKLQVEAGNNQIYLYEYSFVDKDVPEVPHTNIRGATHCAQSMAI<br>LDGKNLTHTSHTDESFATPEFQKMKKTMREIWYNFIKTGKPVPEGSSLPAWPAARANRSPYMSLDQKIELRGVLLE<br>ERTRFWEDIYQKHYLEPVPPPTPPPKPRSEL	563
	**SlitCXE46**	MWKILILLSVVTLGYCDDGEWKQVRTAQGEVRGRKDPAGGLYAFLNIPYAKVPVGTDRFKAPLPAPIWLEPLDAID<br>RGIICMQPPSPLFDTSSKVMQEDCLITNVYVPDTDDKNLPVIVYIHGGAFEIGFGNMMTPKHLVKNKKVIVVNFNY<br>RLGVHGFLCLGTEDAPGNAGLKDQVALLRWVNKNIASFGGNPDDVTIAGYSAGSVSVDLLMLSKSAKGLFNKVILE<br>SGVSVGFIAIQRNPIENAKTYAKMLNFTNVNDFYLMQEFYKSAPMKSMFMSTFLGRKDSTVLFSPCIERRRGNQAF<br>LDESPYNIIKSGKYNKIPMLIGISNMEGSLQMHHVGIWKDSMNTKFSDFLPVDIKFQSNDERDIVSNKIKEFYFGG<br>QPVGEETILAYLDYFGDIMFGYSTLRAVKLHLEAGHKQIYLYEFSFVDDSVPVIPNTNERRAQHCAQTMAVLEGPV<br>DEETLSEEYRNLKSVIREMWSNFAIHGTPVTEDSSLPTWPTTSAGGSPYMSIGKAVEIKESFLGEHGKFWNKIYEK<br>YYSAPVAVAPPPSQAYHNEL	552
	**SlitCXE49**	MWKLVVLLFCCLAIVLGDDSRVVSTAQGPVRGRLLPGGGVYGFYNIPYASAPTGADRYKAPLPPPTWVTIFEAEDR<br>KIICPHRRSGDPMNAREDCLVVNVFVPDTDNTNLPVHIFVHDGAYQFGFGLQKTFTELVRRQNIISVSFNYRLGVH<br>GFLCLGTEAAPGNAGLKDMVALLRWVNTNIASFGGNPDDVTLSSCSAGSGSLDFLTVSSLTDGLFKKVIQESGTVI<br>GAVGAQVDPIQNARNYATVLNFDNVNDFNSLEEFYKNVTIEQLISRTDAILNNEGVTARFGPCVERDLGQEIFISD<br>SPMNLLSRGNYTKVPRMYGFTTMDGSVRLSIFDTWREMMNEKFSDFLPSELHFDSEEVKEQVAQKVKQFYFGDSPV<br>DDHNIVSYLNYNTDVLFAFPMLRTLSTRVGALGDPIYLFEYSFIDENSPVLPQTDENGARHCSQERIVADGDVTAA<br>TNEYKRMQEIMRDYWMNFISNGYPSSSDPSLPTWTPANAQRSPHMSLGRQIVLRDSSPIPERAAFWDELFDEYFRG<br>PIVDMDPVPPTPTPAPGAASSLVMSNIIFVSLLLSFYFTLYLN	575
	**SlitCXE50**	MWKCVLLVFCSILILVQGQDDWLQVNITQGPVRGRKDPNGGLYVFYNIPYATAPTGRDRFKAPLPPPVWMSPLDAV<br>NRNIRCPQPNVLEHTQEDCLVVNLFVPDTTDTNLPVLVFVHGGAFQNGHGLSDQYRELVRTNRLIAVSFNYRIGVH<br>GFLCLGTEGAPGNAGIKDQVALLHWVNTNIAKFGGNPQDVTLAACSAGSGAVDVLTLSSATTGLFQKAILESGSST<br>GAVGVIIDPIQNAKNYAKVLNFDNVDDLDSLEEFYRNAPFQLLTSRVDEIVDNKDTAVRFAPCVERDIGQERVITD<br>APMNVIKRGNYTKVPLLYGFTNMEGAMRLPYFDDWKVQMNEKFSDFLPAELHFDNDEEKEKVAHRVKQFYFGDSDV<br>SEDTMEQFLLYFTDVLFAYPMLKSLSARVEALGDTIYLFEYSFVDEDTPSFPHTNVRGAQHCFQSRAVRDQDLTGR<br>TDAYRRMVQIAREYTLNFILTGSPTSTDARFPLWRPANAQRSPHMSIGPVIELKDDILGDRAAFWDSIYEEHYRGP<br>LVPDGYTGAASSIMISKIIILMAVTVGMLNW	563
	**SlitCXE40**	MQISKIDGNNNKNTELMKASLLFGHANEVCEAEGIMQYRSDKVASVFGEPRVDPLVLISSQGLVMGHRATDGDYSM<br>FLGVPYARVDAADPFGPSREIQPFEEIIFNANDGTVRCPQLGESSIDCLRLNIYVPFKASSSNPLPVLVWIHGGNF<br>VRGSAGDYGVRNLVRHDVVVVTVNYRLGPYGFMCLDTPRVPGNQGLKDQYTALNWIRSNIASFGGNPYNVTISGHD<br>AGATSVLLHMYADNLKLFNKVIVESGTPQSEGMFVNSDENMAIKIAEHLGVNTTDGNEALEFLINSPHNLVAGAAH<br>ELNLPLGPCKEKSFSGIENFVENDPYALTNARKVRNTPVLIGYTSRERDSLSADYFDSDPFYEKLKNNFNLNGQQL<br>ENAATFVRRFYIGDNPVTSDVSTQLQNFESDFVINHPSERIITRLLDENVGSVYEYLFSYTGSTNDGAGHSSELNY<br>LFNDGSGNIVRSEDDQLIADRISTLWTNFVKYGDPTPQTSDLLPVKWTPVTISSRPTMVIDTDIRMDSRVENQRMA<br>FWDLFYSVYGSYSNLARECSFTDCC	557
clade006	**SlitCXE41**	MTSLGRISVALLVLFHHTQCLPRVDPLVDTKVGLIRGLRADDGNYSKFLGIPYAQVNPDNPFGESTPQPKFEETFE<br>AYDESAVCPQWYWWTGEIDGNPDCLQLNVYVPDTANSNNRLPVMVWIYGGAFERGEFSQKLYGPTYLVKHDVIMVA<br>INYRVGPYGFMCLDTPEIAGNQGLKDQHLGLQWVKDNIEAFGGDANQITLFGLSAGGHSIDLHLLSQREVIYNKVI<br>MQSGSSLAATVLYEPDKLAHIKLAQHLGFETTDTNEAISFLAKSDSNLVIAATLDLGIIFKPCVENSFEGVEPFLN<br>SSWIHAGTPKVRNMPVLTGYNEHELASFHYNKDEEYFKNLNIISDYMKRIFNFGEEELQEMSDSIAHFYFGDEAIS<br>SDVKLALIYMDSDFTYIHPIQRSIQKYLEARARNVYYYVFSYLGGRNAILDNGGVEDNSSNVCCAAHADELPYLFN<br>MVNKPPPTEHDEIVIERMTTMWTNFAKYGDPTPQTSDLLPVKWEPLTKESFTYMDIGSELSLGRRPAHDRMAYWDL<br>FYKLNGDKHK	543
	**SlitCXE69**	MWIVGLLVLLCGLVQGLYRVDPLVSTNKGLIRGLRSNEGYAQFLGVPYAVVDKNNPFGPSVPHPGFDDIYEAYESN<br>VCPQVSSDEAIGTIDCLTMNIYVPSAATTQNRLPVMVWIHGGAFVTGSAQTEGNPNFLLRNDVIVVAINYRLGAYG<br>FMCLDIPEVPGNQGLKDQVLALRWINENIEAFGGDVSQITVFGESAGGMSVNLHLLSSYERLFQKAIIQSGPASSP<br>WLIVESDKTIPIKLANFLDFETEDTLEALEYLASVDVHTLIKVAHDLKITDSTGTKNPGTLPCIEKEIDGVESFIT<br>NHPMNINTPKVRNTPVIIGHTNNELPFQYGLADSDFFANYDFSLLSNAFDLEDDWEDAVNDVRHFYIGDEAANKNV<br>ADKITDFASDFVFNHPTQRMADRILDIGGRTVYRYVFSYSGNRNLLKVLFNLNATGAIHADELGYLFDNVELFGEQ<br>VTPQDQLMIDRLTTLWTNFAKYGDPTPATSDLLPVKWQPITKTSQPYLNLDSDLTLGARPFHDRMAFWDLFYKLYG<br>NQQKYGLSGK	542
	**SlitCXE100**	MWSVILLISFCGLVQGLSRVDPLVSTKSGLIRGLLTDSGYAQFLGVPYAEVNKSNPFGPANPHPGFNDIYEAYKSN<br>VCPQVSYNTVVGTIDCLTLNIYVPSAATSKNRLPVMVWIHGGYFIVGSGDAVRIPDYLLRNDVIVVTINYRLGAYG<br>FMCLDIPEVSGNQGLKDQVLALRWINDNIDAFGGDVTQITVFGESAGGMSVNLHLLSSYEPLFQKAIIQSGPAVSP<br>WVMKGTDNSVPIKLAEALNFPTDDVSEAIAFLSTIEVHTLIRLAHDLKLTFFMSLEDSPSTVPCVEKKMEGVEHFL<br>TEYTMTIESTKLKNVPVILGVTSNELFMGYGTADDEFFRNFDFTVLNTAIDLGEKFDEAVEDIRHFYIGDEDTSEK<br>VKDEITNFASDVLFNHPTERMAKRLLELGAKSVYRYVFSYSGGRNVIKIRLNLNGTGAVHADELGYLFDSGELTGD<br>IAPEDQLMIDRITTMWTNFAKYGDPTPSTSGLLPIKWEPITRTTHPYLNLDSDLSLESRPFHSRMAFWDLFYKVYV<br>N	533
Cl	**SlitAchE1**	MRVVLAALTALAARALAGPHEHRARHHAPEHPPHFPAPAPPQPYRGHGEAVRYNPELDTILPRLEDHETSSKRAKF<br>EDAETSSKRAKYDERFYSNHERADEEPMADEPQLGPEDDDPLVVRTRKGRVRGITLTAATGKKVDAWFGIPYAQKP<br>VGDLRFRHPRPAESWGDEILNTTTLPHSCVQIIDTVFGDFPGAMMWNPNTDMQEDCLFINIVTPRPRPKNAAVMLW<br>VFGGGFYSGTATLDVYDAKILVSEEKVVYVSMQYRVASLGFLFFDTPDVPGNAGLFDQLMALQWVKDNIAYFGGNP	695

ade027		HNITLFGESAGAVSVSLHLLSPLSRNLFSQAIMQSGAATAPWAIISREESILRGIRLAEAVHCPHSRTDMGPMIEC<br>LRKKSPDELVNNEWGTLGICEFPFVPIIDGSFLDELPARSLAHQNFKKTNLLMGSNTEEGYYFILYYLTELFPKEE<br>NVGISREQYLQAVRELNPYVNDVGRQAIVFEYTDWLNPDDPIRNRNALDKMVGDYHFTCGVNEMAHRYAETGNNVF<br>TYYYKHRSKNNPWPSWTGVMHADEINYVFGEPLNPGKNYSPEEVEFSKRLMRYWANFARTGNPSINPNGESTKIYW<br>PVHSATGREYLSLAVNSSTVGHGLRVKECAFWQKYLPQLMSATNKPEPPKNCTSSAAPIKVPYEIIGVGVVIATGL<br>AKTTMFKYIV	
	**SlitAchE2**	MISNTKIVFTKLLLCCIVSGAWTRSWANHHDTTTSTTQTTPTTSQAPKNFHNDPLIVETKSGLVKGYAKTVMGREV<br>HIFTGIPFAKPPLGPLRFRKPVPIDPWHGVLEATAMPNSCYQERYEYFPGFEGEEMWNPNTNISEDCLYLNIWVPQ<br>HLRVRHHQDKPLTERPKVPILVWIYGGGYMSGTATLDLYKADIMASSSDVIVASMQYRVGAFGFLYLNKYFSPGSE<br>EAPGNMGLWDQQLAIRWIKDNARAFGGDPELITLFGESAGGGSVSLHMLSPEMKGLFKRGILQSGTLNAPWSWMTG<br>ERAQDIGKVLVDDCNCNSSLLAADPSLVMDCMRGVDAKTISVQQWNSYTGILGFPSAPTVDGVFLPKDPDTMMKEG<br>HFHNTEVLLGSNQDEGTYFLLYDFLDYFEKDGPSFLQREKFLEIVDTIFKDFSKIKREAIVFQYTDWEEITDGYLN<br>QKMIADVVGDYFFVCPTNYFAEVLADSGVEVYYYYFTHRTSTSLWGEWMGVMHGDEMEYVFGHPLNMSLQYHTRER<br>DLAAHIMQSFTRFALTGKPHKPDEKWPLYSRSSPHYYTYTADGTSGPAGPRGPRASACAFWNDFLNKLNELEHMPC<br>DGAVTGPYSSVAGTTLPIVLLTTLATTVAL	638

Table S2: Spodopteran GSTes reconciled nomenclature, with new annotations combined with previous studies.

Spodoptera litura				
This study	Zhang et al., 2016	Cheng et al., 2017	Genbank GCF_002706865.1	chromosome
SlGSTe1	AAS79891	SWUSl0010470 _SlituGST14	LOC111364724	Chr1
SlGSTe2	ACZ73898	SWUSl0064760 _SlituGST05	LOC111351682	Chr14
SlGSTe3	ACZ738999	SWUSl0064770 _SlituGST08	LOC111351684	Chr14
SlGSTe4	KF482955	SWUSl0064780 _SlituGST07	LOC111351681	Chr14
SlGSTe5	KF482956	SWUSl0091750 _SlituGST24	LOC111355027	Chr20
SlGSTe6	KF482957	SWUSl0010470 _SlituGST15	LOC111347873	Chr1
SlGSTe7	KF482958	SWUSl0108040 _SlituGST10	LOC111357178	Chr24
SlGSTe8	KF482959	SWUSl0040720 _SlituGST23	LOC111349356	Chr9
SlGSTe9	KF482960	SWUSl0040700 _SlituGST17	LOC111349355	Chr9
SlGSTe10	KF482961	SWUSl0040720 _SlituGST22	LOC111349356	Chr9
SlGSTe11	KF482962	SWUSl0005590 _SlituGST38	LOC111364883	Chr1
SlGSTe12	KF482963	SWUSl0038410 _SlituGST12	LOC111348828	Chr8
SlGSTe13	KF482964	SWUSl0038420 _SlituGST13	LOC111348827	Chr8
SlGSTe14	KF482965	SWUSl0040710 _SlituGST18	LOC111349355	Chr9
SlGSTe15	KF482966	SWUSl0038400 _SlituGST11	LOC111348844	Chr8
SlGSTe16		SWUSl0040730 _SlituGST16	LOC111349158	Chr9
SlGSTe17		SWUSl0064750 _SlituGST06	LOC111351683	Chr14
SlGSTe18		SWUSl0005600 _SlituGST25	LOC111364904	Chr1
SlGSTe19		SWUSl0040750 _SlituGST21	LOC111349160	Chr9
SlGSTe20		SWUSl0040740 _SlituGST20	LOC111349159	Chr9

Spodoptera frugiperda corn strain

This study	V6 genome assembly (Gimenez et al., 2020)	chromosome
SfCGSTe1	SFRUCORN610000010054	Scaffold_107
SfCGSTe2	SFRUCORN610000007603	Scaffold_116
SfCGSTe3	SFRUCORN610000007603	Scaffold_116
SfCGSTe4	SFRUCORN610000007603	Scaffold_116
SfCGSTe5	SFRUCORN610000003468	Scaffold_118
SfCGSTe6	SFRUCORN610000010054	Scaffold_107
SfCGSTe7	SFRUCORN610000012733	Scaffold_114
SfCGSTe8	SFRUCORN610000013771	Scaffold_47
SfCGSTe9	SFRUCORN610000013772	Scaffold_47
SfCGSTe10	SFRUCORN610000013771	Scaffold_47
SfCGSTe11	SFRUCORN610000018645	Scaffold_107
SfCGSTe12	SFRUCORN610000029704	Scaffold_93
SfCGSTe14	SFRUCORN610000013772	Scaffold_47
SfCGSTe15	SFRUCORN610000029703	Scaffold_93
SfCGSTe16	SFRUCORN610000013771	Scaffold_47
SfCGSTe17	SFRUCORN610000007603	Scaffold_116
SfCGSTe18	SFRUCORN610000018646	Scaffold_107
SfCGSTe20	SFRUCORN610000013771	Scaffold_47
SfCGSTe21	SFRUCORN610000013771	Scaffold_47
Spodoptera frugiperda rice strain		
This study	latest V3 genome assembly (Gouin et al., 2017)	chromosome
SfRGSTe1	SFRURICE0000011418	Contig162
SfRGSTe2	SFRURICE0000005971	Contig39
SfRGSTe3	SFRURICE0000005971	Contig39
SfRGSTe4	SFRURICE0000005971	Contig39
SfRGSTe5	SFRURICE0000026315	Contig2
SfRGSTe6	SFRURICE0000011418	Contig162
SfRGSTe8	SFRURICE0000030251	Contig524
SfRGSTe9-1	SFRURICE0000030253	Contig524
SfRGSTe9-2	SFRURICE0000026789	Contig830
SfRGSTe10-1	SFRURICE0000030251	Contig524
SfRGSTe10-2	SFRURICE0000030251	Contig524
SfRGSTe11	SFRURICE0000014441	Contig697
SfRGSTe12	SFRURICE0000003921	Contig401
SfRGSTe13	SFRURICE0000003921	Contig401
SfRGSTe14	SFRURICE0000026789	Contig830
SfRGSTe15	SFRURICE0000003920	Contig401
SfRGSTe16	SFRURICE0000030251	Contig524
SfRGSTe17	SFRURICE0000005973	Contig39
SfRGSTe18	SFRURICE0000014439	Contig697
SfRGSTe20	SFRURICE0000030251	Contig524
SfRGSTe21	SFRURICE0000030251	Contig524

Sequence retrieval

Spodoptera coding sequences for detoxification genes were retrieved from the latest genomic datasets: for *Spodoptera litura* datasets (Cheng et al., 2017) were combined to the chromosome-level assembly from NCBI (Genbank: GCF_002706865.1); sequences for *Spodoptera frugiperda* Corn strain were from the latest V6 genome assembly (Gimenez et al., 2020) and from Gouin et al., 2017 for the Rice strain (V3); sequences for *Spodoptera exigua* were from the chromosome-level genome sequences from NCBI (GenBank: GCA_011316535.1). Given the absence of genome assembly for *Spdoptera littoralis* candidate detoxification cDNAs were identified from a de novo transcriptome (Poivet et al., 2013). Reciprocal tBLASTn and exonerate (Slater et al., 2005) searches were performed against the different datatsets to generate complete repertoires for all detoxification gene families.

Multiple sequence alignments and phylogenetic analysis

Amino acid sequences were aligned using MAFFT (using L-INS-i option) (Katoh and Standley, 2013) implemented in the Geneious software (http://www.geneious.com, Kearse et al., 2012). Phylogenetic trees were constructed using PhyML (Guindon et al., 2010) based on the best substitution model as determined by the SMS server (Lefort et al., 2017), using Nearest Neighbor Interchange (NNI). Branch supports were estimated by a Bayesian-like transformation of aLRT (aBayes) (Anisimova et al., 2011). A dendrogram was then created, annotated and colored using FigTree software (http://tree.bio.ed.ac.uk/software/figtree/).

Refs

Slater, G. S., & Birney, E. (2005). Automated generation of heuristics for biological sequence comparison. *BMC Bioinformatics, 6*, 31. doi:10.1186/1471-2105-6-31

Gimenez, S., Abdelgaffar, H., Goff, G. L., Hilliou, F., Blanco, C. A., Hanniger, S., . . . Nam, K. (2020). Adaptation by copy number variation increases insecticide resistance in the fall armyworm. *Commun Biol, 3*(1), 664. doi:10.1038/s42003-020-01382-6

Cheng, T., Wu, J., Wu, Y., Chilukuri, R. V., Huang, L., Yamamoto, K., . . . Mita, K. (2017). Genomic adaptation to polyphagy and insecticides in a major East Asian noctuid pest. *Nat Ecol Evol, 1*(11), 1747-1756. doi:10.1038/s41559-017-0314-4

Gouin, A., Bretaudeau, A., Nam, K., Gimenez, S., Aury, J. M., Duvic, B., . . . Fournier, P. (2017). Two genomes of highly polyphagous lepidopteran pests (Spodoptera frugiperda, Noctuidae) with different host-plant ranges. *Sci Rep, 7*(1), 11816. doi:10.1038/s41598-017-10461-4

Poivet, E., Gallot, A., Montagne, N., Glaser, N., Legeai, F., & Jacquin-Joly, E. (2013). A comparison of the olfactory gene repertoires of adults and larvae in the noctuid moth Spodoptera littoralis. *PLoS ONE, 8*(4), e60263. doi:10.1371/journal.pone.0060263

Katoh, K., & Standley, D. M. (2013). MAFFT multiple sequence alignment software version 7: improvements in performance and usability. *Mol Biol Evol, 30*(4), 772-780. doi:10.1093/molbev/mst010

Kearse, M., Moir, R., Wilson, A., Stones-Havas, S., Cheung, M., Sturrock, S., . . . Drummond, A. (2012). Geneious Basic: an integrated and extendable desktop software platform for the organization and analysis of sequence data. *Bioinformatics, 28*(12), 1647-1649. doi:10.1093/bioinformatics/bts199

Guindon, S., Dufayard, J. F., Lefort, V., Anisimova, M., Hordijk, W., & Gascuel, O. (2010). New algorithms and methods to estimate maximum-likelihood phylogenies: assessing the performance of PhyML 3.0. *Syst Biol, 59*(3), 307-321. doi:10.1093/sysbio/syq010

Lefort, V., Longueville, J. E., & Gascuel, O. (2017). SMS: Smart Model Selection in PhyML. *Mol Biol Evol, 34*(9), 2422-2424. doi:10.1093/molbev/msx149

Anisimova, M., Gil, M., Dufayard, J. F., Dessimoz, C., & Gascuel, O. (2011). Survey of branch support methods demonstrates accuracy, power, and robustness of fast likelihood-based approximation schemes. *Syst Biol, 60*(5), 685-699. doi:10.1093/sysbio/syr041
